# How deeply does your mutant sleep? Probing arousal to better understand sleep defects in *Drosophila*

**DOI:** 10.1038/srep08454

**Published:** 2015-02-13

**Authors:** R. Faville, B. Kottler, G. J. Goodhill, P. J. Shaw, B. van Swinderen

**Affiliations:** 1 Queensland Brain Institute, The University of Queensland, Brisbane, Australia; 2 School of Mathematics and Physics, The University of Queensland, Brisbane, Australia; 3 Washington University School of Medicine, St. Louis, Missouri, USA

## Abstract

The fruitfly, Drosophila melanogaster, has become a critical model system for investigating sleep functions. Most studies use duration of inactivity to measure sleep. However, a defining criterion for sleep is decreased behavioral responsiveness to stimuli. Here we introduce the Drosophila ARousal Tracking system (DART), an integrated platform for efficiently tracking and probing arousal levels in animals. This video-based platform delivers positional and locomotion data, behavioral responsiveness to stimuli, sleep intensity measures, and homeostatic regulation effects – all in one combined system. We show how insight into dynamically changing arousal thresholds is crucial for any sleep study in flies. We first find that arousal probing uncovers different sleep intensity profiles among related genetic background strains previously assumed to have equivalent sleep patterns. We then show how sleep duration and sleep intensity can be uncoupled, with distinct manipulations of dopamine function producing opposite effects on sleep duration but similar sleep intensity defects. We conclude by providing a multi-dimensional assessment of combined arousal and locomotion metrics in the mutant and background strains. Our approach opens the door for deeper insights into mechanisms of sleep regulation and provides a new method for investigating the role of different genetic manipulations in controlling sleep and arousal.

A better understanding of the functions of sleep requires better methods to study sleep in model organisms. The discovery over a decade ago that *Drosophila* flies sleep^1^,^2^ has had a profound impact on the sleep field^3^. This is because the powerful genetic tools that have made *Drosophila* a successful model to study neuronal function could now be applied to understanding the functions of sleep. Therefore key hypotheses and theories on the functions of sleep, such as a role in synaptic homeostasis^4^,^5^,^6^ could be investigated in a genetically malleable model organism. In addition, a growing number of research groups are turning to this relatively simple behavioral readout in *Drosophila* to characterize gene functions and neural circuits^7^, separate from any direct investigation of sleep or brain functions. Sleep is likely to recruit a wide diversity of genetic and neuronal pathways that may be more extensive than those controlling circadian rhythms, so sleep measures are highly likely to provide a behavioral phenotype for a broad variety of genetic investigations.

One reason why sleep studies in *Drosophila* have been so successful is because exactly the same paradigms that have been used to monitor diurnal activity levels and circadian rhythms have been adapted to study sleep^8^. Circadian rhythms in *Drosophila* have traditionally been measured using infrared beam interruption devices (Trikinetics, Waltham, MA), where a single beam bisecting a 65 mm tube is sufficient to detect the activity of flies housed individually in tubes (flies tend to walk back and forth the length of the tube, thus breaking the infrared beam once in every crossing). Early sleep studies revealed that five minutes without beam-crossings was associated with prolonged inactivity and increased arousal thresholds, suggesting a sleep-like state^1^. Subsequent studies confirmed that 5 min or more of inactivity was indeed a reliable indicator of sleep in *Drosophila*, at least in wild-type flies^8^,^9^. Almost every *Drosophila* sleep study has thus used this 5 min cutoff as a criterion for quantifying sleep metrics, and most still use infrared activity monitors, although there has been a recent move to video tracking^10^,^11^,^12^.

However, a thorough genetic investigation of sleep requires re-evaluation of arousal thresholds in different strains; behavioral responsiveness levels in different mutants will not necessarily follow the pattern shown by a wild-type strain. In addition, sleep metrics are not restricted only to duration data, but should also consider ongoing changes in behavioral responsiveness and sleep intensity, in order to fully capture sleep functions in different strains. For example, a long-sleeping fly may be sleeping lightly, or a short sleeping fly might be sleeping deeply; activity monitors and simple webcam interfaces cannot distinguish between these possibilities. Indeed, ongoing tracking of animals and active probing of arousal levels with mechanical stimuli has revealed that flies may sleep in distinct intensity stages, and that their arousal levels change throughout the day and night, even within a single sleep bout^13^. A major challenge for the field has been to provide one integrated platform that examines a comprehensive set of sleep metrics, from video tracking to controlling a probe stimulus and testing sleep-deprivation effects, to finally providing processed locomotion, arousal, and sleep intensity data at the end. Ideally, this should be possible using equipment and computational power that is readily available to any *Drosophila* lab. Here, we describe such a platform, the *Drosophila* ARousal Tracking system (DART).

We illustrate the power of DART by examining key measures in the study of *Drosophila* sleep, focusing on a set of genetic variants. We examine two commonly used genetic background strains, as well as two mutants predicted to have opposing sleep phenotypes. Controlling for genetic background effects is a crucial aspect of any *Drosophila* behavioral study. Yet outcrossing to a common genetic background can be a double-edged sword, as this may introduce a distinct set of behavioral effects. This is especially true for sleep studies: for example, except for the Canton S strain, few background strains have been well characterized for arousal thresholds^1^,^9^,^13^. We uncover different arousal thresholds and sleep profiles in the background strains, and we reveal unexpected sleep effects in the mutants that are invisible without actively probing for arousal. Finally, we propose a multi-dimensional scaling approach to better assess how a variety of behavioral readouts together characterize sleep and arousal in different strains. DART therefore also provides a powerful method to map different genetic variants in phenotypic space.

## Results

### Experimental setup and fly tracking

**Figure 1:**
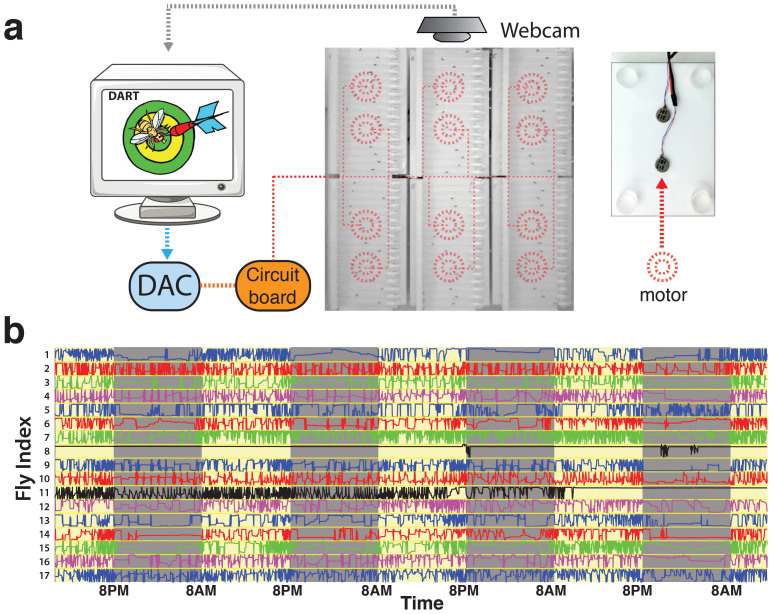
The Drosophila ARousal Tracking (DART) system. (a). Left: DART software records movies via a webcam while simultaneously controlling motor stimuli via a digital to analog converter (DAC). The multiple motors (represented by red dashed circles) are connected to the DAC via a simple circuit board. Middle: Six platforms are positioned in a 3 × 2 arrangement of 17 flies per platform, with two motors per platform. Right: Each platform has two motors glued underneath. The platform has four pedestals (clear circles) that slot into a larger base. (b). Movement traces for 17 flies over four days (yellow background) and nights (grey) outputted from DART. X-axis is time of day and y-axis is horizontal displacement in the tube for each fly. The different colors (blue, red, green purple) are assigned so that the individual traces are easier to distinguish. Graphical user interface supervision allows selection of unusable flies: black traces (flies 8 and 11) have been marked for removal, as flies appeared to have died. Artwork: Benjamin Kottler and Bruno van Swinderen.

Adult virgin female *Drosophila melanogaster* flies were loaded individually into 65 mm glass tubes (Trikinetics) that were plugged at one end with standard yeast-based fly food (see Materials and Methods). Loaded tubes were aligned on a custom-made plastic platform (17 tubes per platform), and multiple platforms (up to 6 at a time) placed on a stage to be filmed (Fig. 1a), in a 25° incubator (Tritech Research). Two shaft-less motors (Precision Microdrives) were glued to the underside of each platform, and vibrations made by these motors were controlled by the DART software interface, via a digital-to-analog convertor (Measurement Computing). The DART interface was also used to record fly activity using a USB-webcam (Logitech) (Fig. 1a; and Materials and Methods). Fly locations were determined by an image subtraction algorithm (Fig. 1b; Fig. S1; see Materials and Methods), and quality control for tracking was performed via a graphical user interface (Fig. 1b; Fig. S1). While our current study is adapted around an established preparation to study sleep in *Drosophila* (single flies in narrow glass chambers), DART will work with any chamber dimensions, different animal sizes, or multiple animals per chamber.

### Sleep tracking

**Figure 2:**
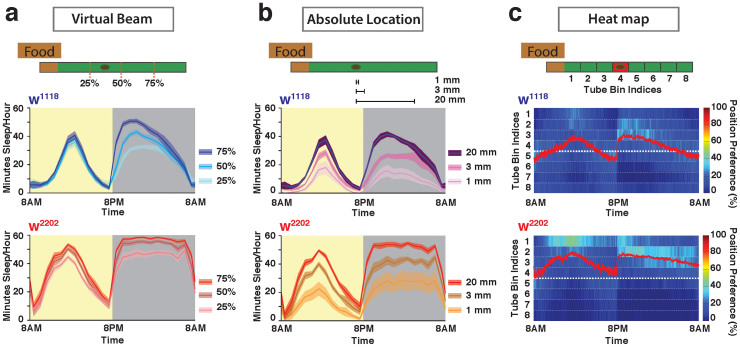
Distinct sleep profiles in two related *white* background strains, w^1118^ and w^2202^. (a). Classical sleep profiles derived from virtual beam-crossing experiments. Flies were tracked continuously, but a virtual “beam” was positioned in three different positions along the 65 mm tubes housing individual flies (25%, 50%, and 75%) to simulate activity readouts from infrared-based tracking systems. Sleep was defined as 5 min or more of inactivity (no beam crossing^1^). The approximate size of the fly is indicated by a dark oval in the tube. (b). Sleep profiles derived from absolute location data, for both *white* strains. Sleep was defined as 5 min or more without movement, which was established for 3 different movement thresholds (1 mm, 3 mm, and 20 mm). The 20 mm movement threshold is essentially equivalent in distance to a beam-crossing experiment, as in a. (c). Heat map of position preferences in the tubes for both *white* strains over multiple days and nights. The individual fly tubes were divided into 8 bins, and position preference (% per unit time over all bins) was plotted as a heat map, with warmer colors indicating a higher probability of flies being in that position. Red line indicates average position for the strain. The thicker white dashed line indicates the mid-point in the tube. See Fig. S2 for additional information on sleep metrics in these strains. N = 45 for w^1118^ and N = 46 for w^2202^. Confidence intervals are SEM.

To see whether our video-based strategy captured basic sleep metrics as traditionally quantified by infrared beam interruption devices^8^, we defined a virtual beam across the center of each tube and counted “beam” crossings per minute (50% position in Fig. 2a, see also Materials and Methods). We tested our system by examining two classical genetic background strains, w^1118^ and w^2202^ (w^1118^ is a white-eyed Canton S strain, and w^2202^ is an isogenized variant of this strain^14^, also known as *isoCJ1*^15^). Most *Drosophila* sleep studies define sleep as five minutes or more without any beam crossing. Implementing this well-established sleep threshold in DART, we found that our video tracking system accurately replicated the sleep profiles and associated metrics from published data using the traditional devices^1^,^8^, although w^1118^ appeared to sleep less than w^2202^ (Fig. 2a, Fig. S2).

Separating each fly tube into equal halves (50%) assumes that activity will be best reflected by the flies' walking along the entire length of the tube and back, which they typically do in this confined context. However, fly activity may not be equally distributed all along the tube length^12^, especially since food distribution is asymmetric (Fig. 2, top schemas). Variability in positioning the tubes in the infrared devices may therefore lead to some variation in sleep and locomotion phenotypes. To test this, we changed the position of the virtual beam, bringing it closer (25%) or further (75%) from the food (Fig. 2a, top schema). We found that variation in the position of this virtual detector of fly activity significantly altered sleep profiles and associated sleep metrics in the two white-eyed, wild-type background strains (Fig. 2a; Fig. S2a, c, e, g). Positioning the beam further away from the food overestimated sleep duration.

Since filming allows continuous and accurate tracking of fly movements, we next determined sleep profiles based on absolute location for these strains (Fig. 2b). We contrasted 3 different movement thresholds, 1 mm (the approximate length of a fly), 3 mm, and 20 mm (equivalent to a beam-crossing experiment), and we kept the sleep criterion at 5 min immobility (although this variable can also be adjusted). Not surprisingly, we found that for both strains a 20 mm movement threshold strongly resembled the mid-position beam crossing profile, and the 1 mm threshold revealed significantly less sleep (Fig. 2b, Fig. S2b, d, f, h), likely due to high-resolution jitter. We therefore chose 3 mm as a suitable threshold for subsequent sleep tracking experiments.

Most animals prefer to sleep in defined locations^16^, and there is some evidence that *Drosophila* flies prefer to sleep near a food source^2^,^12^. Since DART accurately tracks fly positions along the tube, we next investigated whether these were unevenly distributed across multiple days and nights in the two related *white* background strains. We found that fly distribution in the tubes was highly uneven, and that flies indeed remained closer to the food at specified times, especially in the middle of the day and the beginning of the night (Fig. 2c, Fig. S2i, j). However, the two *white* strains behaved differently when tracked this way: while w^1118^ position preferences across 24 hours were more evenly distributed around the tube midpoint, w^2202^ spent most of its time in the half closer to the food. Accordingly, w^2202^ appears to sleep more than w^1118^ when analyzed by single-beam interruption methods (Fig. 2a, Fig. S2). Clearly, other measures are required to ascertain whether w^2202^ indeed sleeps more than w^1118^, because w^2202^ could be remaining closer to the food for other reasons. As a general rule, it appears that the 25% break better matches real-time movement data than the 50% or 75% break, for these strains.

### Arousal probing

Although ongoing locomotion changes and position preferences can be a good predictor of sleep behavior in *Drosophila*, an under-studied method of measuring sleep is to actively probe an animal's responsiveness to stimuli^17^. Sleeping animals should have increased arousal thresholds, compared to awake animals that are simply not moving. Previous work has shown that mechanical stimuli of varying intensities can be used to probe sleep intensity in *Drosophila*^9^,^13^. However, in contrast to the widely applied 5 min inactivity criterion, behavioral responsiveness is not probed in most *Drosophila* sleep studies and no standard methodology exists to quantify behavioral responsiveness in flies. We attached small motors to the underside of the plastic platforms holding the fly tubes^13^, so that we could probe the flies' responsiveness to mechanical stimuli (Fig. 1a); the motors are controlled by the same DART interface, and can be programmed to deliver patterns of stimuli over several days. Although we use motor vibrations in this study, any other device can be used to probe different arousal modalities (e.g., light, sound, odors).

**Figure 3:**
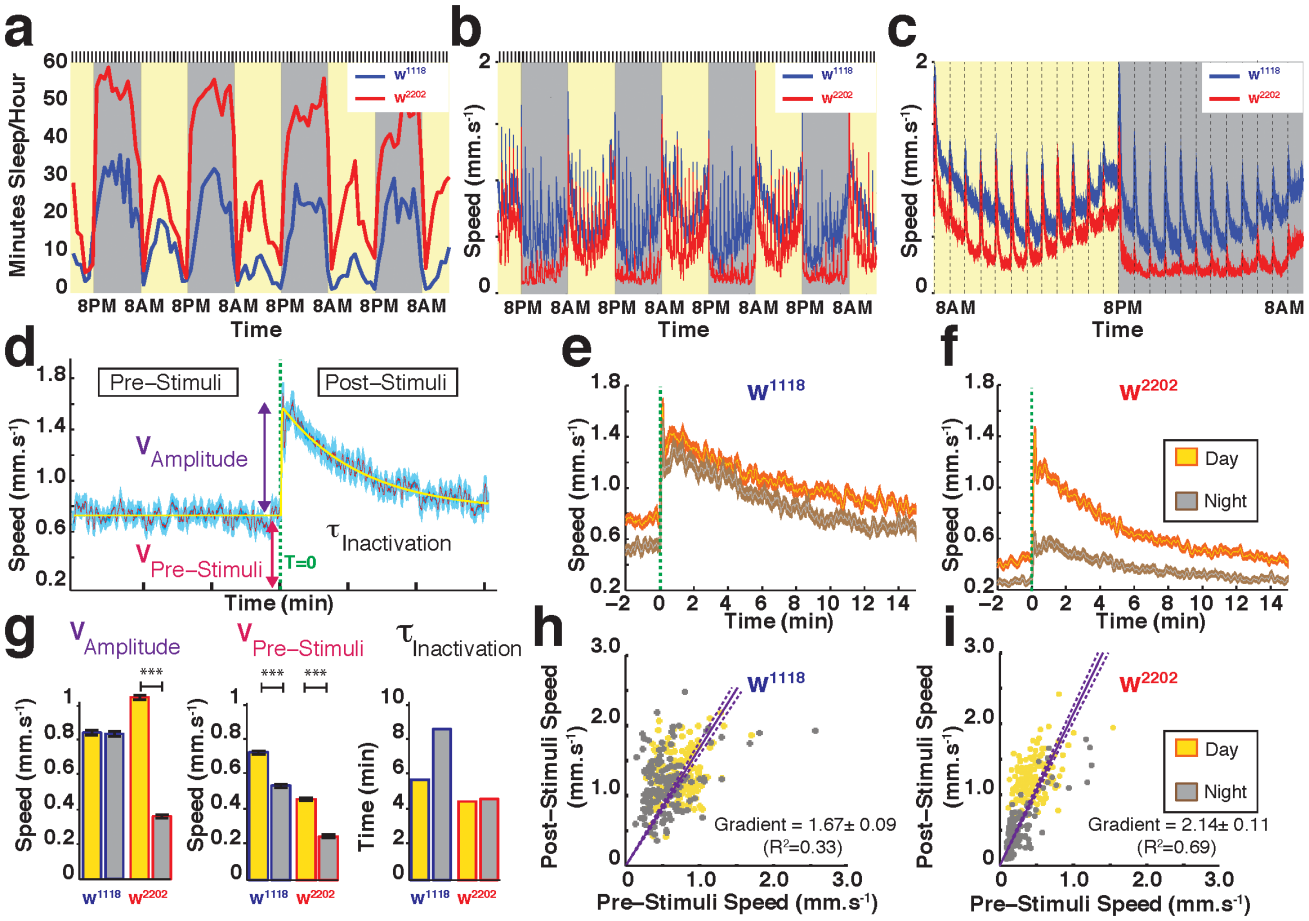
Hourly arousal probing reveals significant differences between *white* strains. (a). Average sleep profiles over several days (yellow) and nights (grey) for w^1118^ (blue) and w^2202^ (red). Minutes of sleep per hour were determined by absolute location data, based on a 3 mm movement threshold as in Fig. 2b. All flies were stimulated hourly (by vibrations, indicated by the black lines above the graph). N = 47 for w^1118^ and N = 50 for w^2202^. (b). Average speed of w^1118^ (blue) and w^2202^ (red) for the same set of flies, over the same time period as in a. The responses to the vibrations (black lines above the graph) are evident as spikes in the speed. Data are averaged for all flies, not only immobile ones. (c). Average speed data for day and night for the same set of flies. The vertical dashed lines indicate hourly vibrations testing for behavioral responsiveness. (d). Characterization of behavioral responsiveness curve. Average speed (purple) (±SEM, light blue) is shown for a wild-type strain, with four metrics derived from a best fit (yellow trace) of the average response. Fitted parameters were: the average speed 1 min before the stimulus (or the baseline speed (V_Pre-Stimuli_), the average speed 1 min after the stimulus (Post-Stimuli Speed), the amplitude of the response (V_Amplitude_), and the time constant of the response (τ_Inactivation_). Green dashed line indicates timing of vibration stimulus (T = 0). Flies analyzed for responsiveness profiles were not necessarily immobile. (e). Average response (±SEM) for w^1118^, for day (yellow) and night (grey). (f). Average response (±SEM) for w^2202^, for day (yellow) and night (grey). (g). Best fitting response parameters (as outlined in d) for w^1118^ and w^2202^ during the day (yellow) and night (grey). N = 2256 responses for w^1118^ and N = 2400 responses for w^2202^, divided equally among 47 and 50 flies, respectively, for day and night. (h). Scatterplot of Pre-Stimuli speed *versus* Post-Stimuli speed, for individual day (yellow dots) and night (grey dots) events in w^1118^. A linear regression of the data (±SEE) is shown, with associated slope and correlation indicated. (i). Scatterplot of Pre-Stimuli speed *versus* Post-Stimuli speed, for individual day (yellow dots) and night (grey dots) events in w^2202^. A regression of the data (±SEE) is shown, with associated slope and correlation indicated.

We first probed arousal thresholds in the two *white* background strains, by delivering stimulus trains of increasing vibration intensity, every hour over several days and nights (Fig. S3a, see Materials and Methods). We found that arousal thresholds in quiescent flies from both strains followed a typical diurnal profile (Fig. S3b), as shown previously for wild-type flies^13^. At night, flies from both strains were more likely to sleep through even the strongest vibrations, resulting in a ceiling effect. Based on these results, we designed a simple protocol to probe behavioral responsiveness every hour over several successive days and nights (Fig. S3c, see Materials and Methods). These hourly probes (a train of five 200 ms pulses set at 1.2 g) decrease sleep slightly, because a subset of sleeping flies will be awoken hourly (data not shown). More importantly, the hourly probes did not alter the distinct sleep profiles shown by the two strains, based on traditional 5 min sleep criteria, with w^2202^ apparently still sleeping more than w^1118^ (Fig. 3a). Also, we found in a previous study that the hourly stimuli do not cause sleep deprivation effects^13^.

We next investigated the flies' responsiveness to the stimuli, to better understand how sleep quality may differ in these strains. Simply examining the flies' locomotion (i.e., speed) revealed important differences between the strains: w^2202^ is clearly slower on average than w^1118^ (Fig. 3b). In addition, w^2202^ displayed clearer day/night differences compared to w^1118^, with strong responses during the day and minimal responsiveness at night. In contrast, responses in w^1118^ seemed equally strong during the night and the day. Closer examination confirmed this observation (Fig. 3c), and also confirmed that baseline speed was consistently greater in w^1118^, day or night, with increasing average speed as the night progressed (Fig. 3c). Thus, both video tracking and arousal probing reveals striking differences between the *white* strains that were not evident using traditional beam-crossing methods.

### The shape of the response

While population measures (as in Fig. S3b) effectively describe behavioral responsiveness in *Drosophila*, another approach to studying arousal levels in flies is to average the response kinematics in a strain, as a fitted curve^18^. Averaging the stimuli response across several days reveals a typical locomotion response curve characterized by several key parameters: the average pre-stimulus speed (V_Pre-Stimuli_), the response amplitude (V_Amplitude_), which represents the difference between the pre-and post-stimuli speeds, and the response decay time constant (τ_Inactivation_) (Fig. 3d, see Materials and Methods). This response averaging approach confirmed major differences between the two background strains: w^1118^ flies are equally responsive during the day and the night, while w^2202^ is significantly more responsive during the day than the night (Fig. 3e–g). Plotting post-stimulus speed against pre-stimulus speed for all the data provides a snapshot of the diurnal responsiveness of a strain (Fig. 3h, i): both strains were responsive to the hourly stimulus (as evident by the positive gradient for both: 1.67 ± 0.09 and 2.14 ± 0.11 for w^1118^ and w^2202^, respectively). However, the separation between daytime and nighttime responses was much better defined in w^2202^ than w^1118^ (Fig. 3h, i, yellow and grey dots). This raises the question of whether sleep intensity is different between the strains, especially at night.

### Sleep intensity

**Figure 4:**
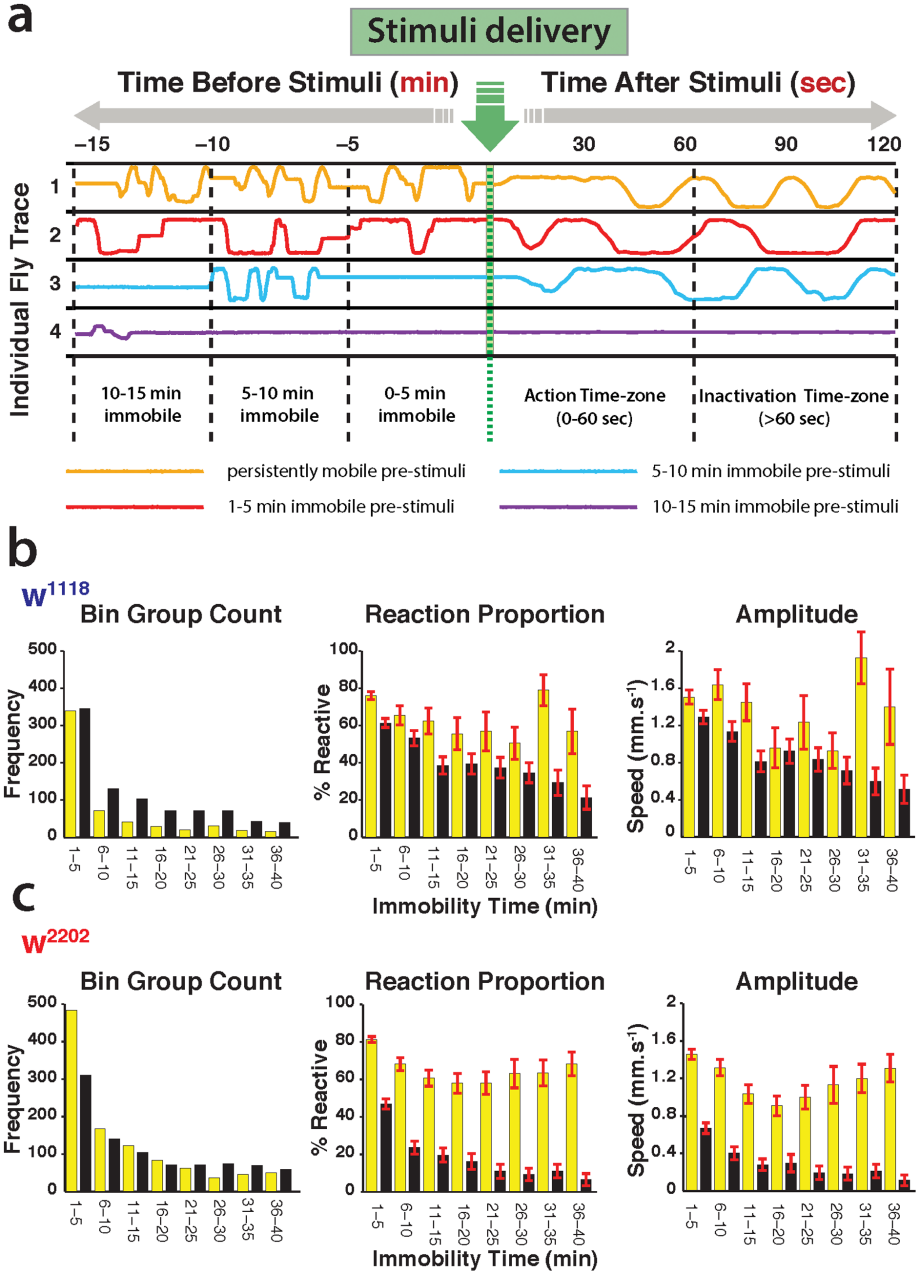
Sleep intensity differs between the *white* strains. (a). Responses were binned according to the duration of prior immobility. Vibration stimuli were delivered once an hour over several days and nights (green arrow). Prior immobility epochs were automatically classified into 5 min bins, as determined by the first retroactive detection of any movement, based on a 3 mm threshold. Four examples are shown, where flies had been immobile for different lengths of time prior to the stimulus (red, blue, and purple lines), and one fly that was moving immediately before the stimulus (gold line). Only immobile flies were included in the subsequent sleep intensity analyses, which summed locomotion responses 1 min after the stimulus (Action time zone). The y-axis represents horizontal fly movement in the tubes. Note that time before and after the stimulus is not on the same scale in the schema. (b). Sleep intensity in w^1118^. Left panel: frequency count for every 5 min immobility bin, for day (yellow) and night (black). Middle panel: the proportion of flies responding (±SEM) for each immobility bin, for day and night. Right panel: the average amplitude of the response for each immobility bin (average speed ± SEM), for day and night. (c). The same sleep intensity calculations were performed for w^2202^ N = 47 for w^1118^ and N = 50 for w^2202^. See Tables S1–4 for corresponding statistics.

Sleep in *Drosophila* is not uniform; wild-type (Canton S) flies sleep deeper during the night than during the day^13^, even though extended bouts of inactivity are prevalent during the day and night^1^. Even within a sleep bout, sleep intensity levels have been shown to vary depending on how long the flies have been immobile^13^. Inactivity bout duration and the number of sleep bouts per unit time reveal some aspects of sleep consolidation^8^,^9^, but such duration-based metrics remain insufficient to address the question of sleep intensity. To measure sleep intensity, responsiveness levels in sleeping flies must be acutely probed throughout a sleep bout, and correlated with how long flies have been asleep. Since the DART platform tracks fly behavior continuously (and all movies are saved), it is therefore straightforward to classify flies into different temporal bins depending on how long they were completely immobile prior to a mechanical stimulus (Fig. 4a). In our dataset for both *white* strains, most flies were immobile for less than 5 min prior to the hourly stimulus, but some were immobile for up to 40 min (Fig. 4b, c, left panels). Binning the data by prior immobility allowed us to ask the question: is there a relationship between how long a strain has been asleep and its average behavioral responsiveness? And, is one strain sleeping more deeply on average than the other?

We found that the proportion of flies responding to the stimulus decreased gradually in the first 15 min of immobility in both *white* strains, day and night (Fig. 4b, c; middle panels), which is indicative of increasing sleep depth. However, daytime sleep became lighter in both strains as immobility bouts lengthened, while nighttime sleep continued to become deeper with time immobile. In general, the average amplitude of the response (as in Fig. 3d) mirrored the population responsiveness proportion (Fig. 4b, c, right panels; Fig. S4). It is clear from these analyses that w^2202^ is sleeping more deeply than w^1118^ during the night, i.e., it is less responsive (see Tables S1–4 for statistics). During the day, both strains sleep equally lightly. Thus, sleep intensity differences are much more clearly partitioned between day and night in w^2202^, compared to w^1118^, and sleep is also deeper at night in w^2202^.

### Sleep disruption

**Figure 5:**
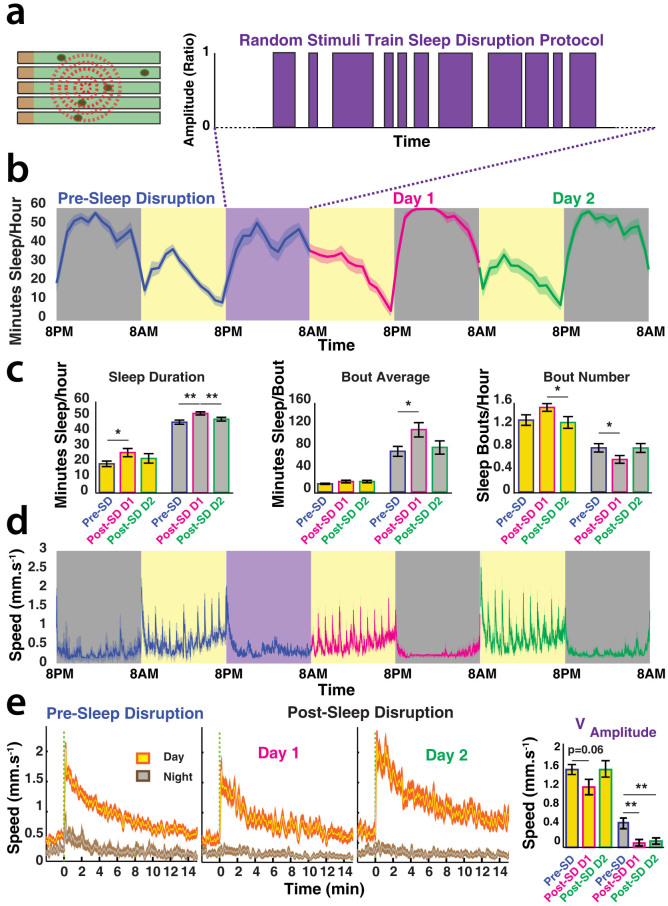
Sleep rebound following a random stimulation protocol. (a). Random patterns of vibration stimuli (set at 1.2 g) were delivered for 12 hours of night. (b). Average hourly sleep duration (±SEM) in w^2202^ before (blue), during (purple box), and after (magenta and green) the random stimulation. Yellow background indicates day, grey is night. (c). Traditional sleep duration metrics (±SEM) for the data from b, in the same color scheme. *, *p* < 0.05; **, *P* < 0.01, by pair-wise 2-tailed *t*-test. (d). Average speed (±SEM) for the same flies as in b. (e). Average daytime and nighttime behavioral responsiveness (±SEM) for baseline and two successive days following the random stimulation. Velocity data (V_amplitude_) are summarized in the panel on the right, for the same three days. **, *P* < 0.01, by pair-wise 2-tailed t-test. Yellow plots represent daytime responses whereas grey plots are nighttime responses; blue surrounding indicates the day before the random stimulation, magenta surrounding is one day after the random stimulation, and green surrounding is two days after random stimulation. N = 17 flies.

Sleep is under homeostatic control, and some of the best evidence for sleep need in any animal is determined by measuring a sleep rebound following sleep deprivation^19^,^20^. A sleep rebound should then be followed by a return to normal behavioral performance, indicating that the sleep deprivation methods did not physically damage the animal. Achieving the right level of gentle handling to interfere with sleep yet not cause damage or undue stress is difficult, and previous *Drosophila* sleep studies have used automated, motorized devices that jolt or rattle the flies awake^9^,^19^. Since the DART platform is already equipped with computer-controlled motors to test behavioral responsiveness, we programmed these to deliver random stimuli trains during 12 hours of night (Fig. 5a, purple box; Fig. S5a, and see Materials and Methods). We found that this gentle, unpredictable stimulus pattern produced a sleep rebound the following day and the following night (Fig. 5b, c; magenta plots). Interestingly, traditional sleep duration metrics were not significantly altered during the random stimulation (Fig. S5b, c). This suggests that it was sleep quality that was compromised, rather than simply sleep duration. Such sleep disruption effects (without affecting sleep duration) can also lead to behavioral deficits^21^. Nevertheless, behavior was still clearly affected during the stimulation protocol: average walking speed was higher and fly location in the tubes was more variable compared to baseline (Fig. S5c, d).

After a day and night of rebound, sleep returned to baseline levels by the second day following the random stimulation (Fig. 5b, c; green plots). Closer examination of behavioral responsiveness before and after sleep disruption also revealed a homeostatic effect for this measure of arousal: the average amplitude of the response to the vibrations was diminished the first day following sleep disruption, but returned to baseline the second day (Fig. 5d, e). Interestingly, nighttime responsiveness was completely abolished following sleep disruption, and remained significantly lower for two days (Fig. 5d, e). This suggests that these homeostatic-sleep processes (responsiveness) are not equivalently distributed between day and night sleep.

### Sleep mutants

**Figure 6:**
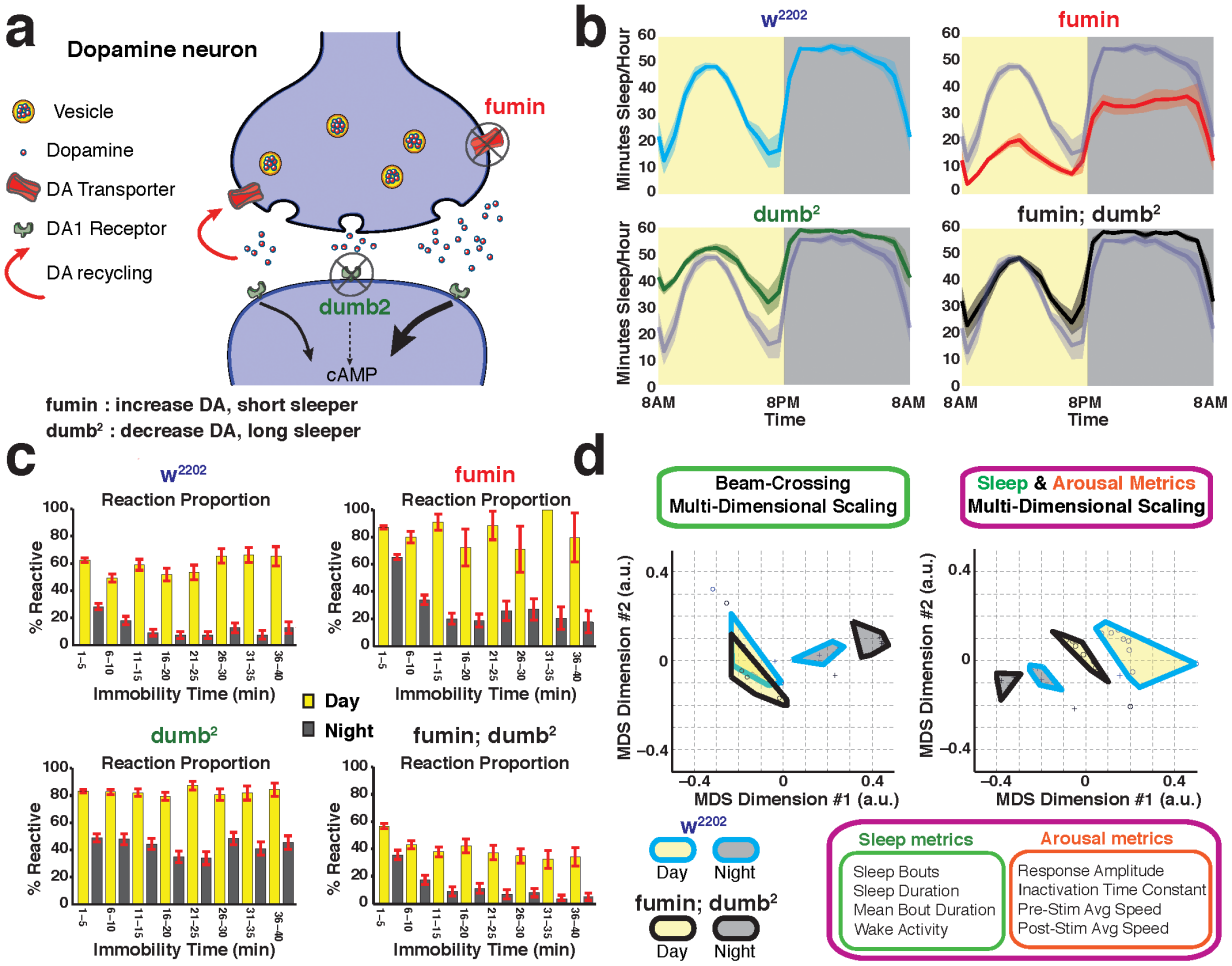
Sleep and arousal measures can be dissociated with DART. (a). Opposing effects of *fumin* and *dumb*^*2*^ on dopamine (DA) function and sleep. Released DA impacts DA1 receptors to initiate cAMP signaling pathways (left black arrow) before being recycled back into the cell (red arrow). *fumin* is mutant for the DA transporter, leading to increased DA levels in the synapse, and consequently increases cAMP signaling in post-synaptic neurons (right thick black arrow). *dumb*^*2*^ is mutant for the DA1 receptor, leading to decreased cAMP signaling (dashed black arrow). (b). Classical beam-crossing sleep profiles (min sleep/hour (±SEM)) based on a 5 min inactivity criterion, for the w^2202^ background strain (blue), *fumin* (red), *dumb*^*2*^ (green), and the double mutant *fumin*; *dumb*^*2*^ (black). N = 68 for w^2202^; N = 67 for *fumin*; N = 62 for *dumb*^*2*^; N = 66 for *fumin*;*dumb*^*2*^. The w^2202^ profile is shown in grey for comparison in the three mutant panels. (c). Sleep intensity profiles (% reactive ± SEM) for the same four strains as in b. (d). Multidimensional scaling (MDS) was used to project the data into a two-dimensional space for easier visualization of the multidimensional relationships between different strains. MDS analyses were performed for *fumin*; *dumb*^*2*^ (blue border) compared to its genetic background strain, w^2202^ (black border), for daytime (yellow) and nighttime (grey) metrics. Left panel: four beam-crossing metrics (as for b) were used in combination for MDS comparing both strains. The different metrics used are indicated in the green box (bottom of the panel). The daytime effects overlap while the nighttime effects are distinct. Right panel: four arousal-based metrics were combined with four beam-crossing sleep metrics (indicated by the larger magenta box) for MDS analyses, resulting in a complete separation between day and night effects in both strains. a.u., arbitrary units.

To test the DART platform on existing *Drosophila* sleep mutants, we contrasted two mutations in the dopamine pathway that have opposing effects on sleep, *fumin* and *dumb*^*2*^ (Fig. 6a). The *fumin* (insomniac in Japanese) strain contains a mutation in the dopamine transporter gene (dDAT), leading to an increase in dopamine at the synapse, which has been shown to decrease sleep^22^. *dumb*^*2*^ is a hypomorphic allele of the dDA1 receptor (also called DopR), which impairs arousal systems in flies^23^, and has been shown to increase sleep^14^. Both mutations were outcrossed to the w^2202^ genetic background, to ensure that any arousal-related phenotypes were specific to each genetic lesion. Using virtual beam-crossing metrics, we confirmed the published sleep phenotypes for these mutants (Fig. 6b). Also consistent with previously published work, combining both mutations in the same strain “rescued” the sleep defects of either mutant (Fig. 6b), presumably because the effects of increased dopamine at the synapse (in *fumin*) are balanced by defective postsynaptic DopR1 function (in *dumb*^*2*^)^14^.

We next investigated whether these DA mutants also displayed different levels of behavioral responsiveness. Simple fly tracking confirmed that *fumin* flies move significantly more than *dumb*^*2*^ flies, day or night (Fig. S6a–c). Both *fumin* and *dumb*^*2*^ responded robustly to hourly mechanical vibrations during the day, but also during the night (Fig. S6d–f). While increased responsiveness in *fumin* is consistent with increased dopamine in this strain, this same result for *dumb*^*2*^ (which has compromised DA signaling) was surprising. Nevertheless, although both mutants respond more strongly than w^2202^ (their common genetic background), their baseline arousal profiles are predictably different from each other, with *fumin* generally more active than *dumb*^*2*^ (Fig. S6g–i).

To better understand the effect of these opposing DA manipulations on sleep, we examined their sleep intensity. Separating the sleep intensity profiles for day and night in these strains yielded a striking result: both mutants remained highly responsive during the day, regardless of how long they had been immobile (Fig. 6c, Fig. S7). This suggests that neither *fumin* nor *dumb*^*2*^ mutants are ever sleeping deeply during the day. Indeed, their average responsiveness level (~80%) throughout the day may suggest that instead both mutants are always awake during the day, even if they are quiescent. During the night, *fumin* sleep is also lighter than in the control strain (Fig. 6c, Fig. S7), as expected due to increased synaptic dopamine in this mutant^14^. Surprisingly, *dumb*^*2*^ nighttime sleep is also lighter, even though dopaminergic function is impaired in this mutant^23^ (Fig. 6c, Fig. S7). The net effect of either dopaminergic manipulation is therefore lighter sleep, day or night, regardless of opposite effects on sleep duration. Combining the mutations (in *fumin*; *dumb*^*2*^ double mutants) appears to “rescue” nighttime sleep intensity, but also increases daytime sleep intensity (Fig. 6c). Thus, combining the two mutations unexpectedly results in deeper sleep, even though each mutation alone produced lighter sleep.

### Multidimensional scaling of arousal data

Using DART, a fly strain can be characterized by many locomotion-related metrics, from counting beam-crossings to fitting responsiveness profiles, and in many instances simple activity-based measures may be sufficient to characterize molecular genetic effects in a mutant strain. However, a global assessment of arousal measures in a strain may help provide an overall picture of how one strain compares to another, and whether “rescue” experiments, for example, only relate to some measures but not others. To provide such a global assessment of locomotion and arousal-related measures, we incorporated a multidimensional scaling (MDS, Fig. S8; see Materials and Methods) option in DART, where selected measures could be assessed in combination to provide a more global picture of behavioral effects. MDS is an exploratory data analysis technique which projects the absolute differences in data points from high-dimensional (Fig. S8a–c) to low-dimensional space (Fig. S8d). This enables visualization of the data structure within high-dimensional space, which in turn can reveal underlying patterns, such as the presence of clusters. Applying MDS on eight combined measures (sleep bouts per hour, total sleep duration, sleep bout duration, waking activity, response amplitude, pre-stimulus speed, post-stimulus speed, decay time constant – as in Fig. 6d) to the two genetic background strains supported the view that day/night activity was different for w^2202^, but not for w^1118^. (Fig. S9a). MDS analysis on the mutants *fumin* and *dumb*^*2*^, on the other hand, supports the suggestion that each mutant occupies a distinct phenotypic space, day and night (Fig. S9b). Such visualization techniques provide a valuable starting point for assessing the effect of subsequent genetic manipulations.

We next used MDS to examine the extent of phenotypic rescue in the double mutant. Previous work using beam-crossing methods predict that the double mutant, *fumin;* *dumb*^*2*^, reverts to wild-type behavior, because opposing dopaminergic effects are combined^14^. While classical sleep metrics on un-stimulated flies indeed suggest phenotypic rescue (Fig. 6b), actively probing the flies with vibration stimuli (Fig. S10) revealed only a partial rescue, as suggested by MDS on the same data (Fig. 6d, left panel: nighttime measures do not overlap). MDS of more arousal metrics, combining beam crossing and responsiveness measures, suggests a further separation between *fumin;dumb*^*2*^ and the w^2202^ background strain (Fig. 6d, right panel). A global assessment of all these measures therefore provides greater insight into phenotypic differences between the two strains, highlighting which measures might be affected by a genetic manipulation, and which remain unaffected. In this specific genetic manipulation, it appears that while sleep phenotypes are indeed rescued (Fig. 6b, c), average speed (day or night) remains lower in *fumin;dumb*^*2*^, compared to the genetic background control (Fig. S10), and responsiveness in general (day or night) remains lower (Fig. S10). This lack of correspondence between sleep duration data and arousal metrics is illustrated by the clear separation between the strains shown by MDS of all eight measures combined (Fig. 6d, right panel).

## Discussion

The discovery that flies sleep has revolutionized sleep research, largely because *Drosophila* molecular genetic tools could be efficiently deployed to address various hypotheses relevant to sleep function. We introduce here the *Drosophila* ARousal Tracking (DART) system, which is a video-based platform that effectively provides well-established activity metrics along with crucial behavioral responsiveness measures to more thoroughly describe sleep, arousal, and locomotion in a strain. DART provides a variety of arousal metrics for *Drosophila*, from activity levels and position preferences, to behavioral responsiveness, sleep intensity, and homeostatic effects. Data can be processed, analyzed, and visualized entirely within DART graphical user-interfaces, or extracted as text files for use in other programs.

We used DART to first compare behavior in two classic *white* strains, which are often used as wild-type backgrounds for genetic studies. Indeed, w^2202^ (also called *isoCJ1*) is an isogenized variant of w^1118^, which was derived from the Canton-S wild-type strain^15^. Surprisingly, these related background strains were found to be quite different for a wide range of sleep and locomotion phenotypes, highlighting the fact that even similar “wild-type” backgrounds might be significantly different. Idiosyncrasies in each strain, such as the propensity for w^2202^ to remain near a food source, are likely to cloud any genetic investigations without access to positional information. Similarly, our finding that w^1118^ responds equally well to mechanical vibrations during the night as during the day suggests decreased arousal thresholds at night in this classic background strain, compared to w^2202^. Outcrossing a mutation to a w^1118^ background might consequently lower arousal thresholds at night, and thereby alter sleep phenotypes (and potentially any other phenotypes regulated by sleep functions). In contrast, outcrossing to w^2202^ would more likely produce flies with a stronger day/night sleep dichotomy, possibly imbuing mutants with the distinct sleep functions that might be associated with light versus deep sleep.

That different forms of sleep exist in mammals and birds is well known^16^. It is becoming apparent that different forms of sleep are also likely in insects^13^,^25^. Some fly strains, such as Canton S^13^ and w^2202^ (this study) sleep significantly more lightly during the day, even though prolonged daytime inactivity, especially around mid-day, might suggest otherwise. What is the function, if any, of lighter daytime “sleep”, and how is it different than nighttime sleep? One possibility may be that different sleep functions, such as memory consolidation and synaptic homeostasis, might be segregated by day and night in insects. However, it is interesting to observe that nighttime sleep in flies also includes epochs of comparatively lighter sleep. This observation could only be made by actively probing behavioral responsiveness in immobile flies, and then plotting the population responses as sleep intensity curves by retroactively binning flies into different immobility duration groups (Fig. 4), which DART does automatically. Finally, it remains possible that lighter sleep in flies accomplishes the same functions as deeper sleep, but maybe to a lesser degree; flies may need to be more alert during the day for obvious ethological reasons. In any case, the DART sleep intensity measures now allow these important aspects of sleep to be addressed in wild type and mutant strains.

As proof of principle, we therefore applied DART to two known sleep mutants with predicted effects on sleep need. *fumin* decreases sleep duration in flies, and *dumb*^*2*^ increases sleep^14^, and these opposing phenotypes as a consequence of defects in well-understood molecules have been convincingly used to understand the role of dopamine in promoting arousal in *Drosophila*^14^,^26^. We placed *fumin* and *dumb*^*2*^ in a w^2202^ background, to preserve the day/night dichotomy more evident in this background. While these mutants behaved as expected for inactivity-based sleep profiles (Fig. 6b), their responses to probing stimuli were very different from those expected: DART revealed both mutants to be more responsive than wild type while quiescent, day or night. This suggests that, to achieve wild-type responsiveness profiles, the level of dopamine signaling has to be maintained within an optimal range. Increased or decreased dopamine signaling leads to equivalent sleep intensity defects, which is consistent with the “inverted U” hypothesis of dopamine function^27^, and with a review of several other dopamine manipulations^28^: most dopaminergic manipulations seem to increase behavioral responsiveness, regardless of whether dopamine function is enhanced or impaired. This observation is also consistent with a recent study showing increased visual responsiveness in flies that have experienced an altered dopamine environment during development, in a *Drosophila* model for schizophrenia^29^. It will be interesting to see whether sleep duration and sleep intensity can be dissociated by altering dopamine levels in different dopamine sub-clusters^30^. Our arousal-based approach predicts that there will be distinct dopaminergic effects on sleep duration and responsiveness, because combining *fumin* and *dumb*^*2*^ rescued one activity measure but not others. This result is consistent with other *Drosophila* studies suggesting that sleep and general arousal are controlled by non-overlapping dopaminergic circuits in different brain structures^18^. Finally, the lack of any significant changes in sleep intensity during the day in both dopamine mutants (Fig. 6c) suggests a potential biological basis for the regulation of arousal thresholds as a function of prior immobility. It is possible that dopaminergic signaling regulates behavioral responsiveness specifically during the day, and that another neuromodulator regulates responsiveness at night. Interestingly, daytime arousal threshold dynamics are rescued when *fumin* and *dumb*^*2*^ are combined (Fig. 6d), although these do not exactly phenocopy the daytime sleep intensity dynamics seen in the common genetic background strain.

The concept of phenotypic rescue is central to genetic analysis; genetic manipulations disrupt a phenotype, and other manipulations are done to restore the phenotype, thus providing an understanding of the underlying mechanisms. Half a century ago, this working concept was first applied to understanding the genetic underpinnings of different behaviors, such as circadian rhythms, courtship, and learning, and genes such as *period*, *fruitless*, and *dunce* were identified and since then slotted into a growing body of knowledge about nervous system function^31^. Sleep is a relative newcomer to *Drosophila* behavior genetics^1^,^2^, and already dozens of genes have been identified as key to the regulation of sleep, as well as a variety of distinct circuits and molecular pathways^3^,^32^. Sleep and arousal in *Drosophila* can be characterized by several sub-phenotypes: total sleep duration, daytime sleep, night time sleep, sleep bout number (day or night), bout length (day or night), activity per waking minute, as well as the novel metrics we have presented here. Clearly, many measures can be grouped as behavioral syndromes, and these should change (or be “rescued”) together, while others are irrelevant to the problem being investigated. Multidimensional scaling (MDS) of multiple phenotypes, as we have shown via the DART platform, provides one way of more thoroughly investigating behavioral rescue or testing hypotheses, even for studies not directly linked to sleep and arousal. We envision that the DART platform could therefore also be effectively used more generally for characterizing different genetic effects in behavioral-phenotypic space.

## Methods

### Fly stocks and rearing conditions

Flies were raised in plastic bottles (Genesee Scientific) at 22°C on standard yeast-based *Drosophila* media, on a 12 hour light-12 hour dark (LD) rhythm. Virgin females were collected under CO_2_ anesthesia, and then aged for 3–5 days in groups of 20–50 flies in plastic vials yeast based agar fly media. For all experiments, individual flies were aspirated into 65 mm glass tubes (Trikinetics, Waltham, MA) containing fly media on one end and sealed with a cotton plug on the other end. Two *white* strains were compared: w^1118^ and w^2202^. These are standard white-eyed, wild-type background strains, typically used as controls for transgenic experiments. w^2202^ is an isogenized variant of w^1118^, also known as *isoCJ1*^15^. *fumin* and *dumb*^*2*^ were kindly provided by K. Kume. These were outcrossed at least six generations to a w^2202^ genetic background^14^.

### Behaviorial platform

Flies were individually housed in 65 mm glass tubes (Trikinetics, Waltham, MA), 17 tubes on a plastic tray (14 × 8 cm), 2–6 trays per filmed experiment. Flies were recorded continuously in avi or mp4 format at 5 frames per second throughout multi-day experiments, as set by the DART software (see below), using a USB-webcam (Logitech) fitted with a wide-angle lens (Zeiss). The DART interface also controlled vibration stimuli delivered to the flies. Shaft-less vibrating motors (Precision Microdrives™; model 312–101) were glued underneath each tray of 17 flies (2 motors per tray), and these were controlled by modulating voltage output with DART, by interfacing with the analogue output channels of a USB data acquisition device (Measurement Computing; 1280 LS). The unit g is used to express vibration amplitude (1 g equals the gravitational force at the surface of the earth, 9.8 m/s^2^). The Precision Microdrive motors have a linear relationship between input voltage and vibration, supporting a maximum voltage of 3.5 V or 2.4 g^13^. The vibration motors were operated at a stimulation frequency of 50 Hz for all experiments. Arousal probing experiments were typically 4–5 days long, with the first day and night discarded to allow for the flies to acclimatize to the tubes.

### Fly tracking and video image segmentation

The DART experiment suite is a graphical user interface (GUI) based program that was developed using Matlab (Mathworks). The DART framework provides the mechanisms by which to A) custom-design stimuli-based experimental protocols, B) track the fly locations from the recorded video files, and C) combine and quantitatively analyze the flies' activity using established *Drosophila* sleep/activity metrics. Guidelines are available in the DART instruction manual, provided at http://web.qbi.uq.edu.au/dart/index.html.

The locations of the flies from the avi/mp4 videos are calculated in a semi-automatic fashion via an image subtraction method that uses an estimate of the image background that is specific to each recorded video. This method is preferred to image subtraction using a static reference image^11^ because the image pixel intensity can fluctuate significantly over the duration of a multi-day experiment. The background image estimate is determined by first reading a stack of 10–20 images that are selected randomly from the candidate video. For each image within the stack, fly locations are determined within manually defined tube regions by calculating the cross-correlation of the raw image compliment with an empirically-defined 2D fly template image. The two types of template image distributions used in DART are the 2D Boltzmann and Gaussian distributions, which take the following functional form:
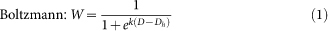

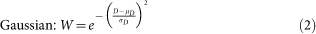
where 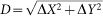, *k*/*D_h_* are the slope factor and half-inactivation distance, and *μ_D_/σ_D_* are the mean/standard deviation distances.

After determining the fly regions over all the background image estimate stack, image regions not occupied by the flies are stored for each image frame. From this, the weighted average of the non-occupied image regions is used to represent the background image. The location of the flies for the remaining frames in the video are then calculated from the subtracted image residual with the most likely (brightest) region within each tube representing the fly's location. The program reads the video into sub-image stacks for each apparatus (50 frames/stack) and calculates the fly locations from the image subtraction residuals (see Fig. S1a–h for a summary of the processes described above). All fly locations were tracked at a rate of 1 Hz, which is consistent with other video tracking methods^12^. Tracking false positives, usually signified by large jumps in fly location, can be fixed manually in post-processing using the DART fly tracking GUI. Flies that have severe tracking issues, or are deceased, can be confirmed and removed via visual inspection through the DART data combination GUI.

### Analysis tools

#### Arousal thresholds

Arousal thresholds were tested with sequentially increasing vibration intensities, from 0 to 1.2 g, in 0.3 g (200 ms) increments, every 15 s, once an hour over 24 hours. Arousal thresholds were calculated by assigning the vibration intensity (g) that evoked a locomotion response (walking at least half the length of the glass tube) in quiescent animals (i.e., flies that had not shown any movement in the preceding minute), and determining the distribution of g values for a strain^13^.

### Elementary Analysis Calculations

#### Absolute Location Kinematic Calculations

Due to the dimensions of the fly tubes, we can approximate fly location spatially by 1 dimension (i.e., the x-location of the fly within the tube), which, for the *i*^th^ movie frame, is denoted by *X_i_*. Therefore, the total distance travelled by an individual fly over *N* movie frames is calculated as follows:
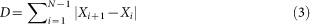
From this, the average speed travelled by a fly over this time duration calculated by:
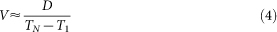
where *T_i_* denotes the time stamp (given in seconds) of the *i*^th^ movie frame from the start of the experiment. The mean population distance/speed, over the given time period, is calculated as the average of the individual kinematic metrics over all members within the population. It should be noted that all kinematics for this study were calculated using this method, unless otherwise explicitly stated.

#### Movement Detection

Consider a fly that at a time, *T*_0_, has a location of *X*_0_. A fly is considered to have moved if, for a given time period, Δ*T*, the distance range is greater than the movement distance threshold, *D_Move_*:
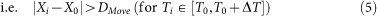
Conversely, the duration of inactivity of an individual fly is calculated as the total time, from an initial time *T*_0_, that the distance range of the fly is less than *D_Move_*:
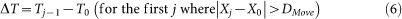


#### Virtual Beam Kinematic Calculations

To recreate the virtual beam kinetics from the DART video tracking, an ideal beam is placed within a given tube at a location, *X_Beam_*, which is calculated as:
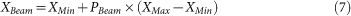
where *X_Min_/X_Max_* is the minimum/maximum x-locations over all flies within a single apparatus tray, and *P_Beam_* is the proportional extent of the tube taken from the left (*X_Min_*) side of the tube (i.e., *P_Beam_* = 0.5 ideally represents the midline of the tube). For a given fly, beam crossings are determined by first classifying the location of the fly with respect to *X_Beam_*, which is given by:
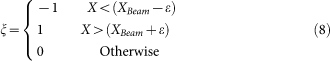
where ε = 1 mm represents a small “dead region” that must be traversed for a true beam crossing to be registered. The reason for this is to prevent registering of false negatives that could occur with random jitter of the fly's location. After applying eqn (8) to all tracked movie frames, all frames where ξ = 0 are replaced by the last preceding non-zero value. From this, all time points where ξ either changes from −1 to 1 (crossing from left to right) or from 1 to −1 (crossing from right to left) are classified as beam crossings.

The virtual beam kinematics equivalent for eqns (3) and (4) is to replace the absolute distance travelled within the specified time period by the number of beam crossings. With regards to detecting fly movement, eqn (5) can be replaced by determining if there was at least one beam crossing within the time period. It should be noted that within DART the virtual-beam midline crossing method is only applicable for the Population movement, and Population sleep/waking analysis functions (see below).

### Analysis Functions

#### Population sleep and waking metrics

In order to determine whether a fly is in a sleeping or waking state, the movement state for each individual fly is calculated for each minute of the day starting at *T_Day0_* (i.e., calculating eqn (5) with Δ*T* = 60 s). For each fly, all bouts of inactivity (i.e., contiguous time bins of inactivity) are determined. If an inactivity bout is 5 min or more in duration (or a “sleep bout”), then the time bins within the inactivity bouts are considered to be “sleep” minutes. All other time bins are therefore considered to be “waking” minutes. Given that the sleep/wake state of each fly is known for each minute in relation to the start of the day, beginning at *T_Day0_* = 8 am, then it is possible to calculate the following population metrics for each hour in the day:

- **Sleep bouts/hour** – Average number of sleep bouts for each individual fly.
- **Sleep minutes/hour** – The average sleep duration for each individual fly.
- **Sleep minutes/bout** –Sleep minutes/hour divided by sleep bouts/hour.
- **Hourly waking speed** – Average of eqn (4) calculated for all waking minutes within the fly population.

To compare variations in the population activity for specific time periods during the day (i.e., day vs night), the aforementioned hourly metrics can be averaged over a duration of *T_GrpDay_* hours, where:
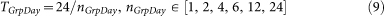


#### Population movement

The population movement analysis is similar to the population waking metric analysis in that the function quantifies fly activity over an entire day. However, the population movement analysis differs in that A) it takes the activity of all flies into account (regardless of their state), and B) the time resolution is much finer.

To calculate this metric, the day is split up temporally into time bins of duration, *T_Move_*. For a given time bin, the average speed of an individual fly is calculated using eqn (4) (with Δ*T* = *T_Move_*), which in turn is averaged over all flies to obtain the population average. This can be extended to multi-day experiments to calculate daily average speed whereby the aforementioned time bins are temporally aligned to *T_Day0_*, and then averaged over all days.

#### Fly location heatmap

The fly location heatmap is calculated by discretising the tube length spatially into *N_HM_* equal regions. From this, the spatial bin location of an individual fly, for any given movie frame, is calculated as follows:
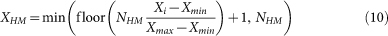
where floor(*Y*) is a mathematical operator which rounds *Y* down to the nearest integer, and *X_min_*/*X_max_* denotes the individual flies' minimum/maximum tube locations, respectively, over the entire experiment. The final population heatmap is calculated by splitting up the experiment temporally into time bins of duration *T_HM_*. Each heatmap column therefore represents the spatial bin location histogram count divided by the total histogram count for a given time bin. From this, the mean fly population location, for a given time bin, is calculated by:
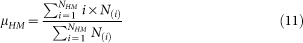
where *N*_(*i*)_ denotes histogram count for the *i*^th^ tube spatial region.

#### Stimuli Response Curve Fitting

The stimuli response curves are formed by first retrieving the fly location traces for the time points *T_Before_*/*T_After_* minutes before/after each stimuli event, and interpolating the fly locations to the nearest second (see Fig. 3d). The stimuli response speed for each individual fly, at the *j*^th^ signal time point, is estimated by eqn (4) from the preceding *N_Avg_* time points:
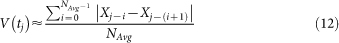
with the population average stimuli response speed being the average over all flies. The relative stimuli response signal is calculated from the absolute stimuli response by subtracting the pre-stimuli speed, which is the average speed of the time points preceding the stimuli event (i.e., −*T_Before_* < *t* < 0). To quantify the features of the stimuli response, the relative speed signals can be fit with either the single-inactivation exponential equation:
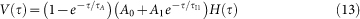
or the double-inactivation exponential equation:
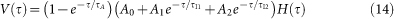
where *τ* = *t* − *δt*, *H*(*t*) is the heaviside step function (= 1 if *t* > 0, otherwise 0), *A*_0–2_ are scale factors *τ_A_* is the activation time constant, and *τ_A_*/*τ_B_* are inactivation time constants. The exponential equation parameters (the scale factors, time constants and *δt*) for (13) and (14) are fitted using the Matlab optimization function, *lsqcurvefit*. For this study, all stimuli response signals were characterized using the single-inactivation exponential equation functional form.

Note that the stimuli response signals can also be grouped by time regions within the day (similar to the Sleep/Waking Metrics). It should be appreciated that if the number of samples in a given time group is too small (approx. < 50) then the exponential form of the response signal will divulge into a noisy signal (for examples, see Fig. S4 & S7).

#### Pre- and post-stimuli speed comparison

The pre/post-stimuli average speeds (*V_Pre_*/*V_Post_*), for each fly, are calculated from eqn (4) for the time domains *T_Pre_*/*T_Post_* minutes before/after each stimuli event. These values can also be grouped by time within the day in the same manner as Stimuli Response Curve Fitting (see above). The response gradient quantifies the effect the stimuli events have on fly speed, and is calculated as the ratio of the post-stimuli to pre-stimuli speeds. The overall response gradient, confidence intervals, and correlation values are all calculated by the Matlab curve fitting function, *fit* using the linear functional form, ‘y = mx'.

In addition to the response gradient, the pre/post-stimuli speed comparison can also be quantified in terms polar coordinates by the phase angle, *ϕ* and magnitude, *R*, which are calculated as follows:
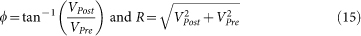


#### Sleep intensity

The immobility time of an individual fly prior to a stimuli event at time, *T_S_*, can be calculated by eqns (5) & (6) using *T_0_* = *T_S_* and reversing the direction of time (i.e. determining the last frame before *T_0_* where eqn (4) is true). These immobility times are grouped into time bins of duration *T_Immob_* (see also Fig. 4a):
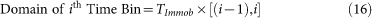
For a given time group that has a total count of *N_Total_* flies, given that *N_React_* flies respond to the stimuli (calculated from eqn (6) using a post-stimuli event response duration of Δ*T* = *T_React_* = 60 s) then the reaction proportion, *P_React_*, is calculated as follows:
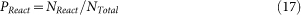
To quantify effect of sleep intensity on arousal threshold, the stimuli response signals are be calculated and quantified for each of the immobility time bins using eqns (12)–(14). Further to this, all of the aforementioned sleep intensity metrics can be differentiated further by separating activity by day/night (where the day/night phases start at 8am/8pm respectively).

#### Multidimensional scaling

Multi-dimensional scaling (MDS) is a data analysis technique that enables the visualisation of similarities/dissimilarities between groups of high-dimensional data in a low-dimensional space^24^. This is accomplished in DART by calculating the following metrics (see Table 1):

- **Sleep Metrics** – Hourly Sleep Bouts, Sleep Duration and Avg Bout Duration
- **Waking Metrics** – Hourly Wake Activity
- **Stimuli Response** – Response Amplitude, Inactivation Time Constant
- **Pre/Post-Stimuli Activity** – Pre-Stimuli Avg Speed, Post-Stimuli Avg Speed

These metrics are calculated for each of the fly background/mutant types, with the metrics being average over the number of user-defined daily time groups (i.e., *n_GrpDay_*_; _see above also). For each individual metric, all the values are combined into a single vector which in turn is used to calculate a normalized 2D square, distance matrix (Fig. S8a–b). The total distance matrix, *Z*, is therefore calculated by summing up these distance matrices over all metrics (Fig. S8c). An MDS algorithm is then applied to *Z* so as to determine the location of an element in lower order M-dimensional space (M = 2 or 3) such that the between-element distances in *Z* are preserved (Fig. S8d).

The MDS algorithms used within DART are Classical MDS (using the Matlab function *cmdscale*) and Non-Classical Metric/Non-Metric MDS (using the Matlab function *mdscale*). For this study, all MDS analysis was calculated using Classical MDS and visualised in 2D space. The justification for using only 2 dimensions to visualise the data comes from the relative MDS eigenvalues, which are less than 5% for all dimensions > 2 (data not shown).

It should be appreciated that MDS is a measure of *relative* similarity/dissimilarity. Therefore, care must be taken when drawing conclusions *between* *different* MDS results. For instance, comparing the results between Fig. S9 (a) and (b) is meaningless because the relative distances between, say, w^1118^ in (a) and *fumin* in (b) have not been accounted for. To directly compare these two fly types using MDS, then they would need to be included in the *same* MDS calculation.

### Sleep disruption

The DART motors were used to disrupt sleep in flies, as well as to probe behavioural responsiveness before and after sleep disruption. To disrupt sleep in flies, a protocol was designed to provide randomized vibration stimuli (set at 1.2 g) throughout the night, from 8pm to 8am. For every stimulus train, a number was drawn from a uniform distribution between the following minima and maxima for 4 parameters: pulse count (4–7), pulse duration (0.5–4 s), inter-pulse delay (0.5–2 s), and inter-stimulus delay (20–40 s).

### Statistical analyses

Statistical significance for the difference between proportion means, for the sleep intensity reaction proportion, was calculated using the pair-wise 2-tailed t-test. For arousal threshold experiments, significant differences in arousal levels were determined using non-parametric Kruskal-Wallis tests. For the sleep intensity reaction proportions, statistical significance was calculated using pair-wise two-tailed z-tests. All other tests of statistical significance of difference means were calculated using a pair-wise 2-tailed t-test.

## Author Contributions

R.F. wrote the DART software. B.K. performed experiments. G.J.G. and P.J.S. provided reagents and analytical tools. R.F., B.K. and B.V.S. conceived the paradigm, analyzed the data, and wrote the manuscript. All authors reviewed the manuscript.

## Supplementary Material

Supplementary Information Supplementary Information

## Figures and Tables

**Table 1:** List of parameters and their association to the analysis functions. The stated parameter values are that which were used to generate analysis results for this study (unless explicitly stated otherwise)

Parameter	Parameter Value	Population Movement	Fly Location Heatmap	Sleep Metrics	Waking Metrics	Stimuli Response Curve Fitting	Pre/Post Stimuli Speed	Sleep Intensity	MD Scaling
***D*_Move_**	3 mm			√	√				√
***T*_Move_**	1 min	√							√
***T*_Day0_**	8:00 AM	√	√	√	√	√	√	√	√
***P*_Beam_**	0.5	√		√	√				
***T*_HM_**	1 min		√						
***N*_HM_**	8		√						
***T*_Before_**	2 min					√		√	√
***T*_After_**	15 min					√		√	√
***N*_Avg_**	10 sec					√		√	√
***T*_React_**	1 min					√		√	√
***T*_Pre_**	1 min						√		√
***T*_Post_**	1 min						√		√
***T*_Immob_**	5 min							√	√
***n*_GrpDay_**	2*			√	√	√	√	√	√

^*^n_GrpDay_ = 24 for all Multi-Dimension Scaling analysis results.
