# Supplementary material for: How deeply does your mutant sleep? Probing arousal to better understand sleep defects in Drosophila
Source: Sci Rep. 2015 Feb 13;5:8454. doi: 10.1038/srep08454 (PMC4326961; doi:10.1038/srep08454)
Supplement: Supplementary Information [file srep08454-s1.pdf]

Supplementary information

How deeply does your mutant sleep? Probing arousal to better understand sleep defects in *Drosophila*.

R. Faville[1]*, B. Kottler[1]*, G.J. Goodhill[1,2], P.J. Shaw[3], B. van Swinderen[1]

1. Queensland Brain Institute, The University of Queensland, Brisbane, Australia
2. School of Mathematics and Physics, The University of Queensland, Brisbane, Australia
3. Washington University School of Medicine, St. Louis, Missouri, USA

* Equal contribution

Email: b.vanswinderen@uq.edu.au

Content (and associated Fig. in main text):

Fig. S1: Image segmentation (Fig. 1).

Fig. S2: Comparison of classical sleep metrics in *white* background strains (Fig. 2).

Fig. S3: Setting the stimulus train (Fig. 3).

Fig. S4: Average response curves (Fig. 4).

Fig. S5: Sleep disruption effects (Fig 5).

Fig. S7: Response curves for sleep intensity for *fumin* and *dumb^2* (Fig. 6).

Fig. S6: Hourly arousal probing for *fumin* and *dumb^2* (Fig. 6).

Fig. S8: Multidimensional scaling (MDS) (Fig. 6).

Fig. S9: MDS of the background strains and dopamine mutants (Fig. 6).

Fig. S10: Hourly arousal probing for w^{2202} and *fumin;dumb^2* (Fig. 6).

Table S1-4: Sleep Probing statistical significance tables (Fig. 4).

Supplementary Figures

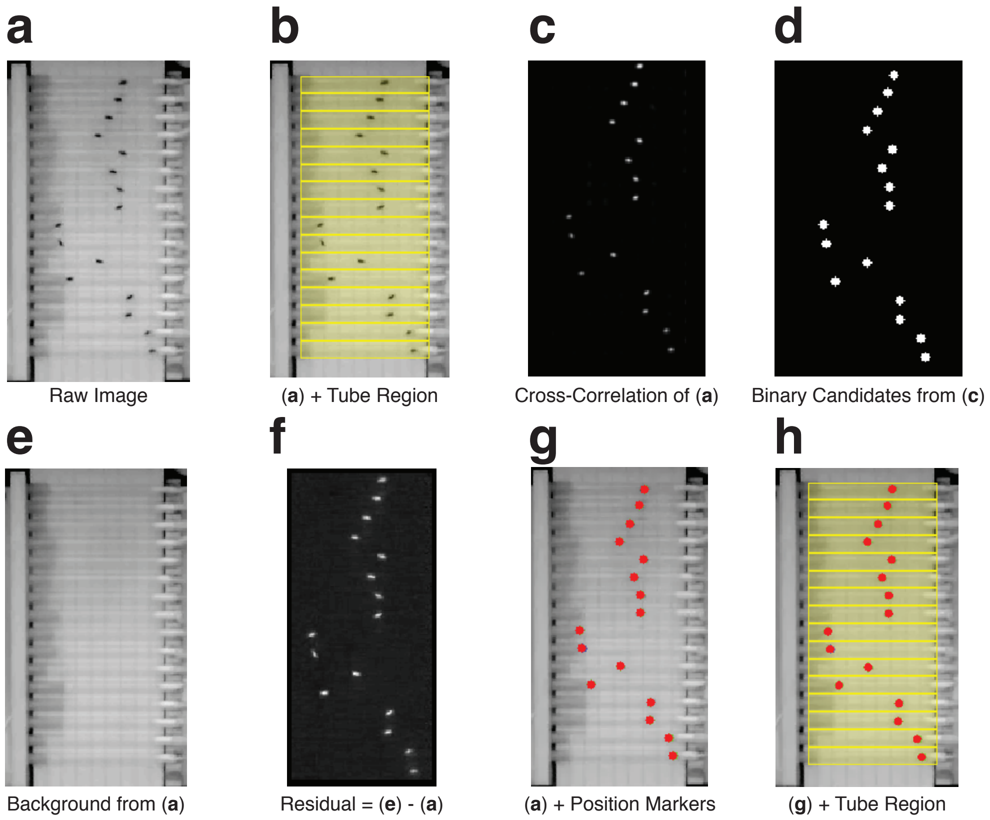

Fig. S1: Image segmentation. **a**. A randomly selected individual movie frame (only one platform is shown). **b.** After manually setting the entire apparatus region where the image processing will occur, tube regions automatically appear (yellow rectangles) specifying each individual fly. These tube regions can be then manually adjusted. **c.** First image processing of the individual frame selected in **a**. A cross-correlation and top-hat transformation characterizing the fly shape is applied on each individual frame (see Materials and Methods). **d.** An estimate of the fly position is then determined after this first filtering process (the cross-correlation in **c**) giving binary candidates on the selected frame. **e.** Over the selected image stack, each frame is processed and the cross-correlation/top-hat transformation applied over the whole image stack. As likely fly regions are calculated, their binary compliment can be used to determine the background image, which is calculated as the weighted average of the non-occupied regions over all the candidate frames. **f.** An image subtraction residual is calculated by subtracting the candidate image from the background estimate. Each white dot highlights the difference between the background and the frame, showing the fly location. **g.** After the segmentation process, position markers can be included over each frame to confirm the fly position over the stack of image. **h.** Tube regions and position markers superimposed over a selected frame.

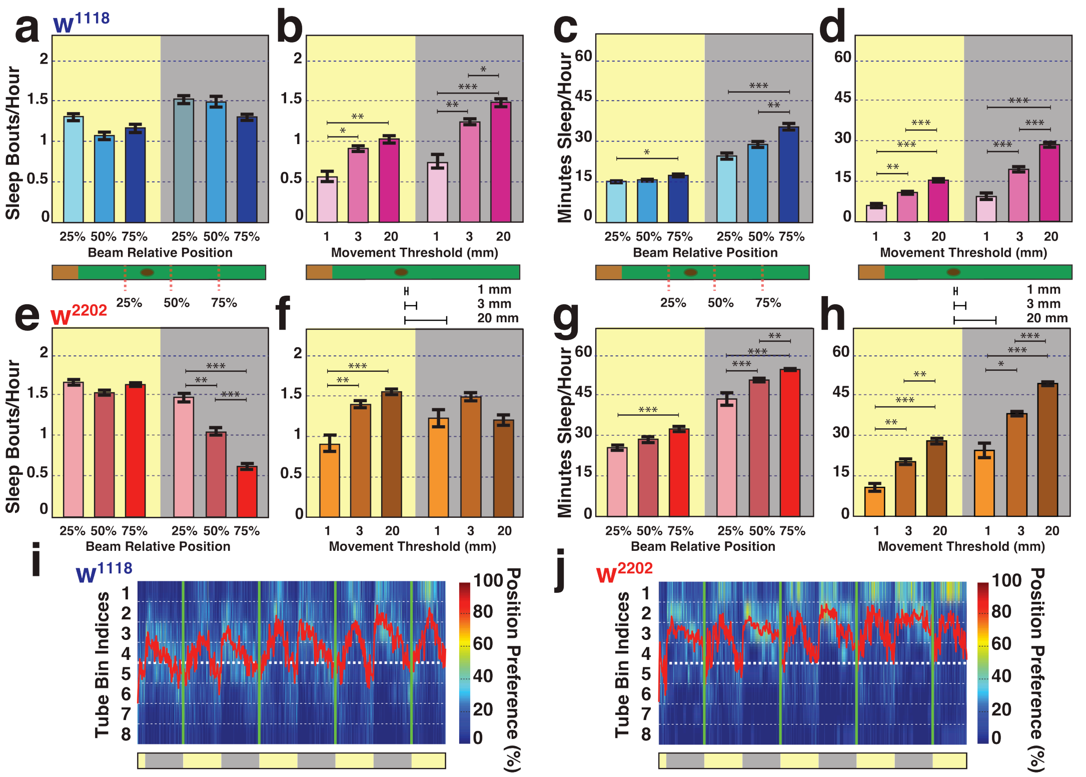

Fig. S2: Comparison of classical sleep metrics in two *white* background strains, w^{1118} and w^{2202} using beam-crossing and absolute location. **a.** Number of sleep bouts per hour for w^{1118} using relative position of the infrared beam. No difference is observed comparing day (yellow) and night (grey). **b**. Number of sleep bout per hour for w^{1118} using absolute location and different movement thresholds. Increasing the movement threshold increases the number of sleep bouts. **c**. Minutes of sleep per hour for w^{1118} using relative position of the infrared beam. Moving the infrared beam further away from the food increases sleep. **d**. Minutes of sleep per hour for w^{1118} using absolute location and different movement thresholds. Increasing the movement threshold increases sleep. **e**. Number of sleep bouts per hour for w^{2202} using relative position of the infrared beam. No difference is observed during the day (yellow) but the number of sleep bouts decreases at night (grey) as the infrared beam is moved away from the food. **f**. Number of sleep bout per hour for w^{2202} using absolute location and different movement thresholds. As the movement threshold increases, the number of sleep bouts per hour also increases. **g**. Minutes of sleep per hour for w^{2202} using relative position of the infrared beam. The further away the infrared beam is from the food the more sleep per hour for day (yellow) and night (grey). **h**. Minutes of sleep per hour for w^{2202} using absolute location and different movement threshold. Greater the movement threshold is associated with longer sleep. *, P<0.05, **, P<0.01, ***, P<0.001, as determined by pair-wise 2-tailed t-test. **i-j**. Heat map over 4 consecutive days for w^{1118} and w^{2202}. Daytime is represented by the yellow boxes underneath the heap map, and the night times by the grey boxes. Green lines represent each day's demarcation. The red line represents the average position of the flies. N=45 for w^{1118} and N=46 for w^{2202}.

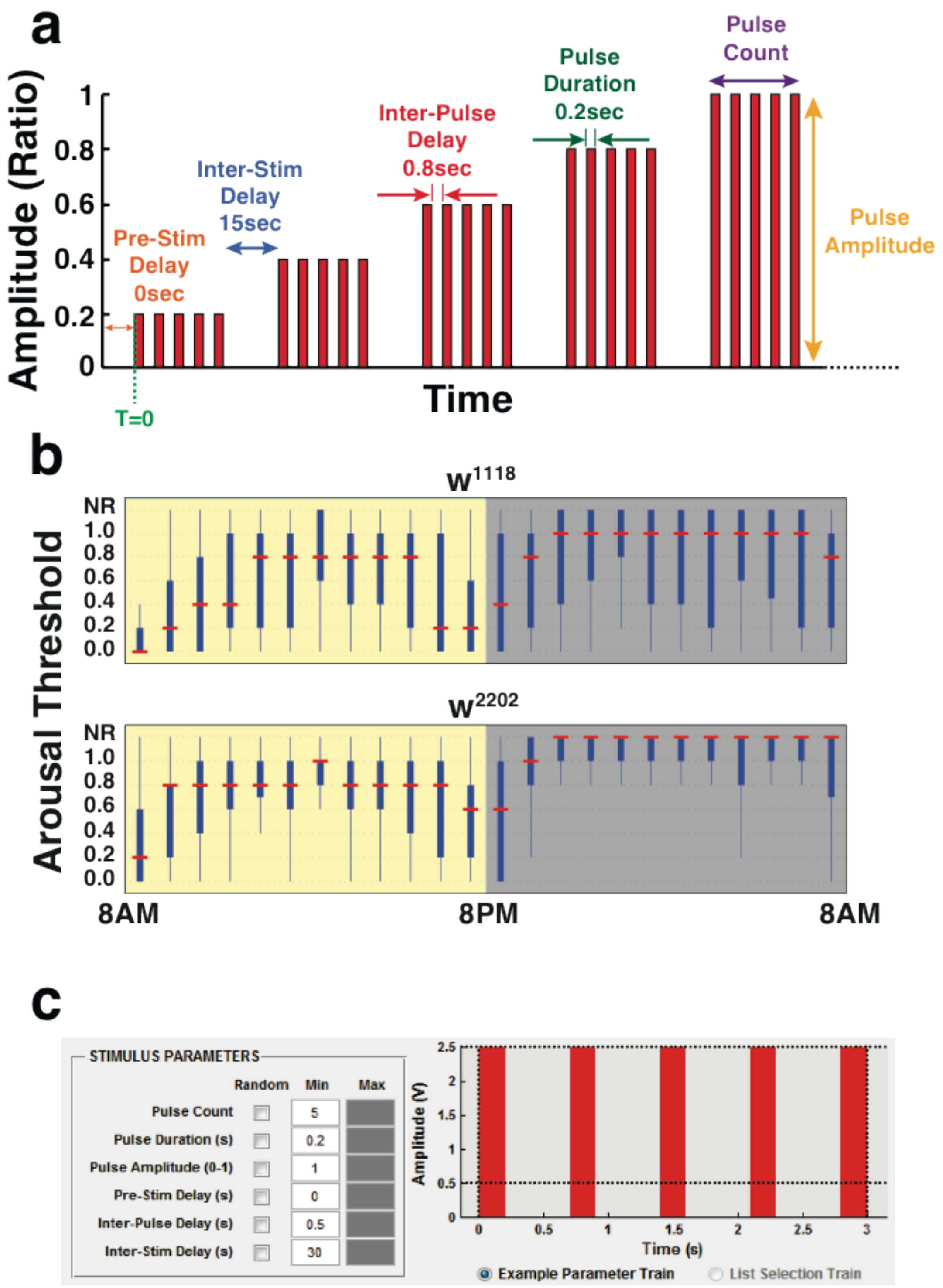

Fig. S3: Setting the stimulus train. **a**. To determine arousal thresholds, stimuli of increasing strength are presented. Each train is five 200ms pulses, separated by 800ms. Inter-stimulus delay is 15s. Pulse amplitude is normalized to 1.2 g. The stimuli train starts at T=0. The different components of the stimuli train are indicated: pre-stimulus delay (orange) which is the delay between the pulse and the beginning of the train (set to 0s), inter-stimulus delay (blue), which is the time between each train (set to 15 seconds), inter-pulse delay (red), which is the time between each pulse within the same train (800 milliseconds), pulse duration (green), which is the duration of each

pulse (set to 200 milliseconds), and pulse count (purple), which is the number of pulses per train (set to 5). **b.** Arousal thresholds were determined hourly across the day (yellow) and night (grey) for w^{1118} (top) and w^{2202} (bottom). Red line=median arousal threshold (amplitude normalized to 1. 2 g), blue bar= 75th percentiles. **c.** DART interface to establish the stimulus train for a sleep probing experiment (5 pulses at 1.2G, delivered hourly, as seen in the final train in a).

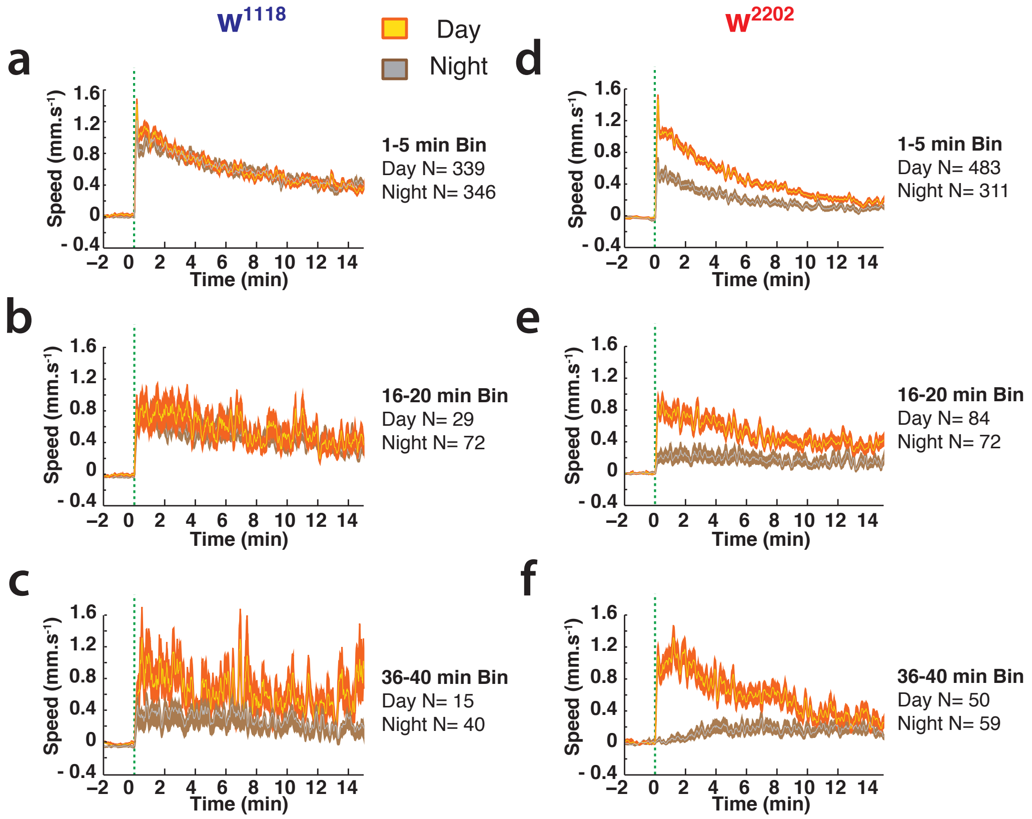

Fig. S4: Average response curves (± SEM) for sleep intensity for w^{1118} and w^{2202}. **a-c.** Examples of the response curves for w^{1118}. **d-f.** Examples of the response curves for w^{2202}. For each set of curves the relevant immobility time bin is indicated. Average responses are represented in yellow (with SEM in orange) for daytime and in grey for nighttime (with SEM in brow). The number of flies included in each bin for day and night is indicated.

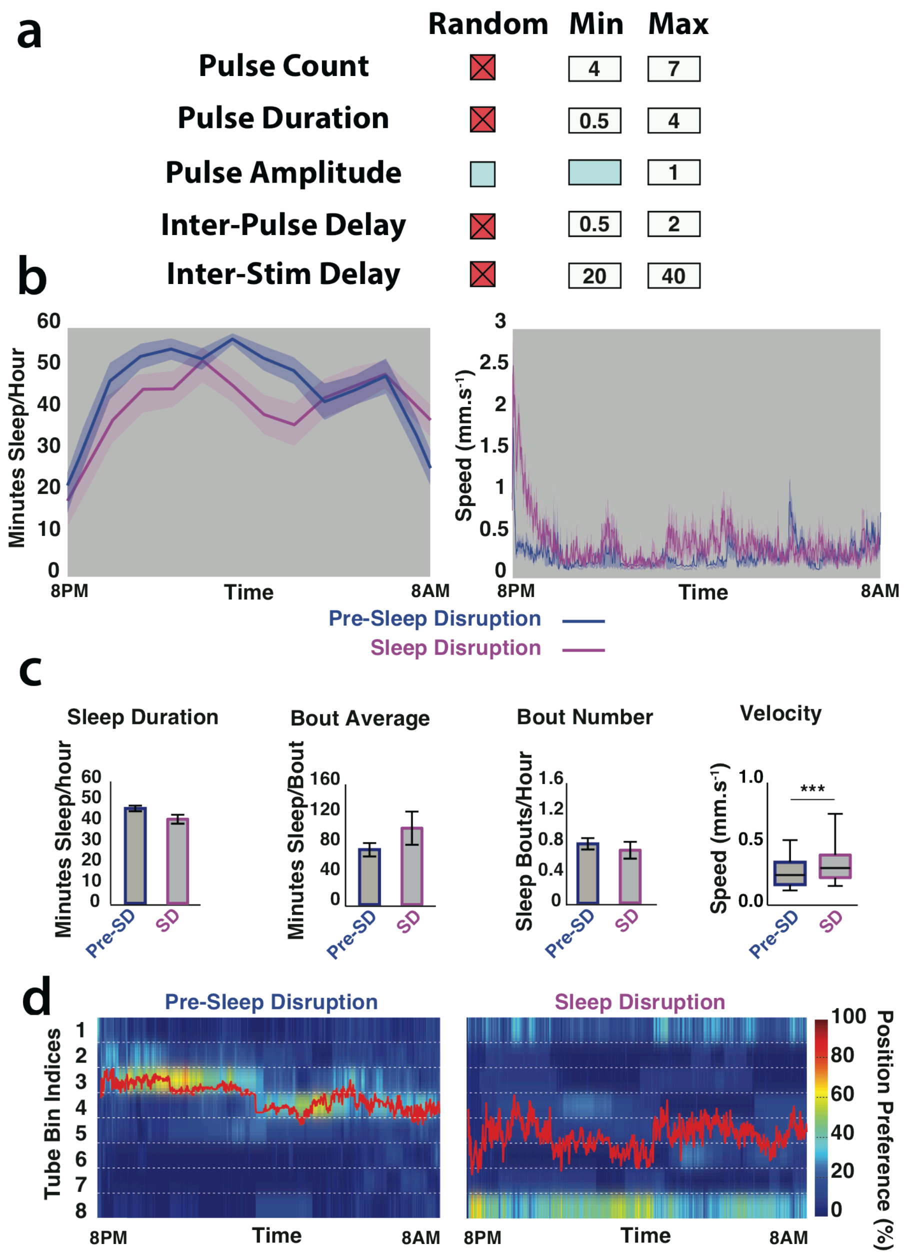

Fig. S5: Sleep disruption effects. **a.** The random stimulus settings in DART (shown in red), with minimum and maximum values indicated for each parameter. Numbers were drawn from a uniform distribution for each parameter. **b.** Hourly sleep duration (± SEM) and velocity (± SEM) for the sleep-disruption night (magenta) compared to baseline (blue). **c.** Classical sleep metrics (± SEM) for flies exposed to the random vibrations (magenta) compared to baseline (blue). Rightmost panel shows velocity differences between these epochs (***, $P<0.001$, Kruskal-Wallis test of medians). **d.** Heatmap position locations during baseline (left) compared to during sleep disruption (right). Average position is shown by the red trace. N=17 flies.

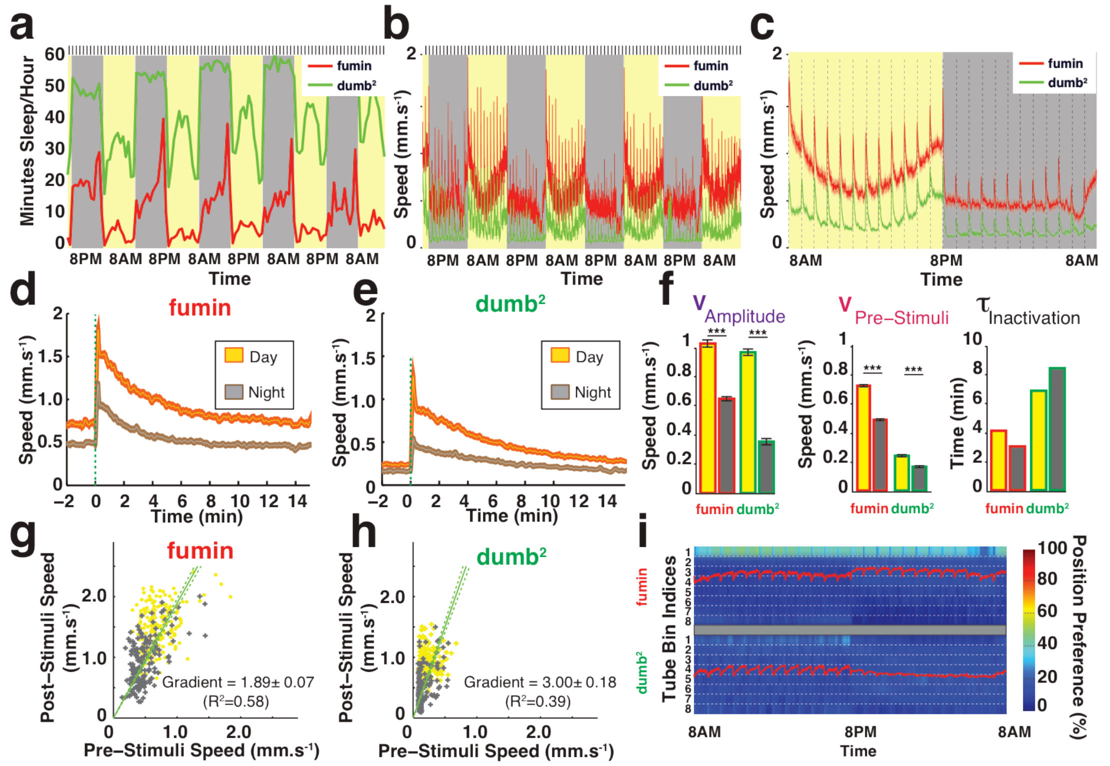

Fig. S6: Hourly arousal probing for *fumin* and *dumb*^{2}. **a.** Average sleep profiles over several days (yellow) and nights (grey) for *fumin* (red) and *dumb*^{2} (green). Minutes of sleep per hour were determined by absolute location data, based on a 3 mm movement threshold as in Fig. 2b. All flies were stimulated hourly (by vibrations, indicated by the black lines above the graph). N=67 for *fumin* and N=62 for *dumb*^{2}. **b.** Average speed of *fumin* (red) and *dumb*^{2} (green) for the same set of flies, over the same time period as in A. The responses to the vibrations (black lines above the graph) are evident as spikes in the speed. **c.** Average speed data for day and night for the same set of flies. The vertical dashed lines indicate hourly vibrations testing for behavioral responsiveness. **d.** Average response (± SEM) for *fumin*, for day (yellow) and night (grey). Green dashed line indicates timing of vibration stimulus (T=0). **e.** Average response (± SEM) for *dumb*^{2}, for day (yellow) and night (grey). Green dashed line indicates timing of vibration stimulus (T=0). **f.** Response metrics for *fumin* (red border) and *dumb*^{2} (green border) during the day (yellow) and night (grey). ***, P<0.001, by pair-wise 2-tailed t-test. **g.** Scatterplot of Pre-Stimuli speed *versus* Post-Stimuli speed, for individual day (yellow dots) and night (grey dots) events in *fumin*. A regression of the data is shown, with associated slope and correlation indicated. **h.** Scatterplot of Pre-Stimuli speed *versus* Post-Stimuli speed, for individual day (yellow dots) and night (grey dots) events in *dumb*^{2}. A regression of the data is shown, with associated slope and correlation indicated. **i.** Heatmap of position preferences for for *fumin* and *dumb*^{2}, with average positions indicated by the red traces.

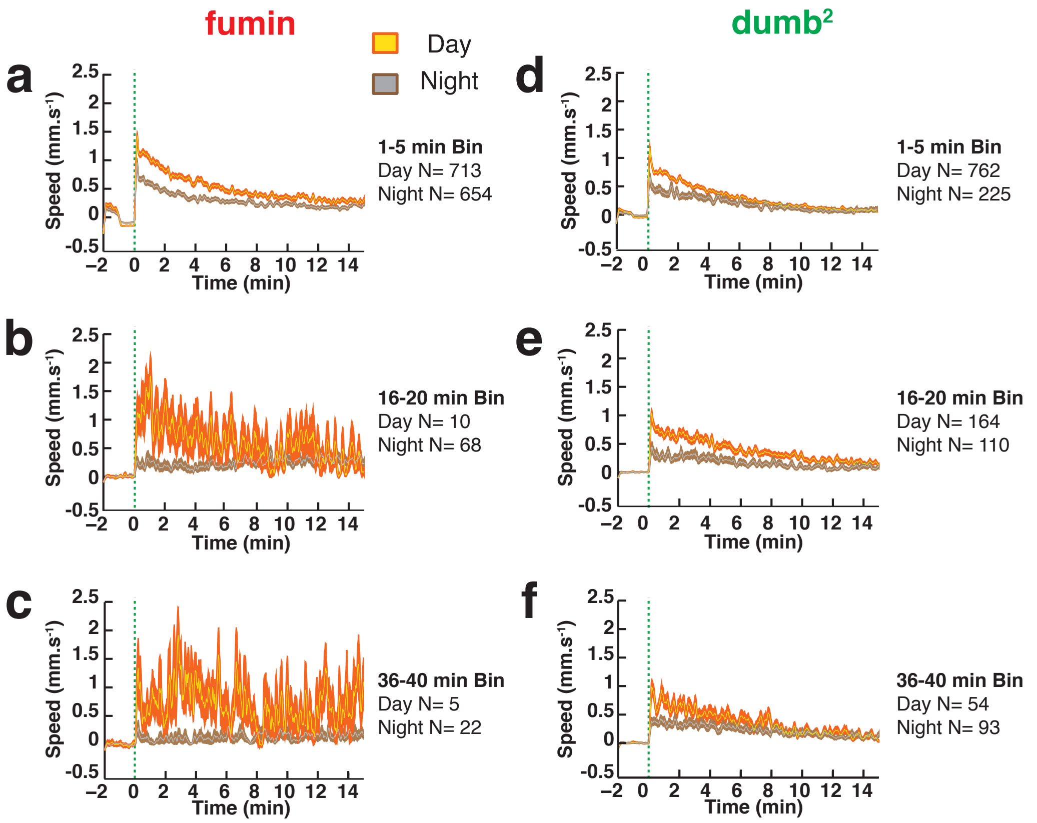

Fig. S7: Response curves for sleep intensity for *fumin* and *dumb*^{2}. **a-c.** Examples of the response curves for *fumin*. **d-f.** Examples of the response curves for *dumb*^{2}. For each set of curves the relevant immobility time bin is indicated. Average responses are represented in yellow for daytime and in grey for nighttime. The number of flies included in each bin for day and night is indicated.

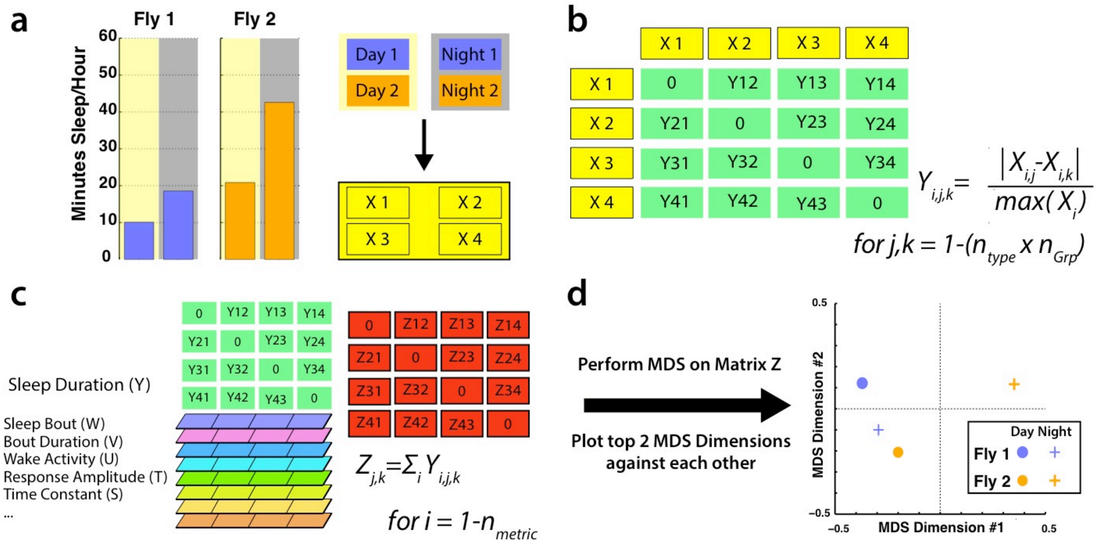

Fig. S8: Multidimensional scaling (MDS). **a.** For a given metric from the MDS metric list, the metric values are calculated over all flies for nGrpDay time groups (which is 2 for this example) and stored in a contiguous vector, **X(i)**. **b.** From this vector, a normalized 2D distance matrix (**Y(i)**) is calculated. **c.** This process is repeated for all the stated metrics, and summed into a final distance matrix, **Z**. **d.** The MDS algorithm applied to Z, which aims to preserve high-dimensional, inter-element distances within the low dimensional space (which for this case is 2 dimensions). Results from this example suggest that nighttime activity for Fly 1 and day-time activity for Fly 2 is the most similar, whereas Fly 2 night-time activity is the most dissimilar out of all the classifications.

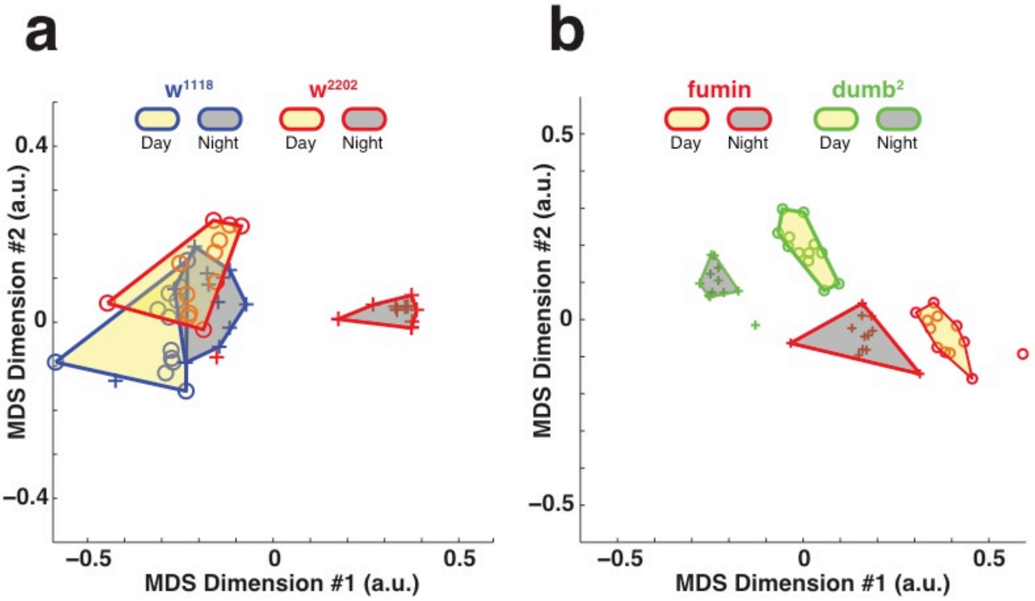

Fig. S9: Multidimensional scaling (MDS) of the genetic background strains and dopamine mutants. **a.** MDS was used to project eight distinct metrics (see Materials and Methods) into a two-dimensional space for easier visualization of the multidimensional relationships between different background strains, w^{1118} (red border), and w^{2202} (blue border) for daytime (yellow) and nighttime (grey) metrics. **b.** MDS was used to project eight distinct metrics (see Materials and Methods) into a two-dimensional space for easier visualization of the multidimensional relationships between the mutant strains (on a common w^{2202} background), *fumin* (red border), and *dumb*^{2} (green border) for daytime (yellow) and nighttime (grey) metrics. a.u., arbitrary units.

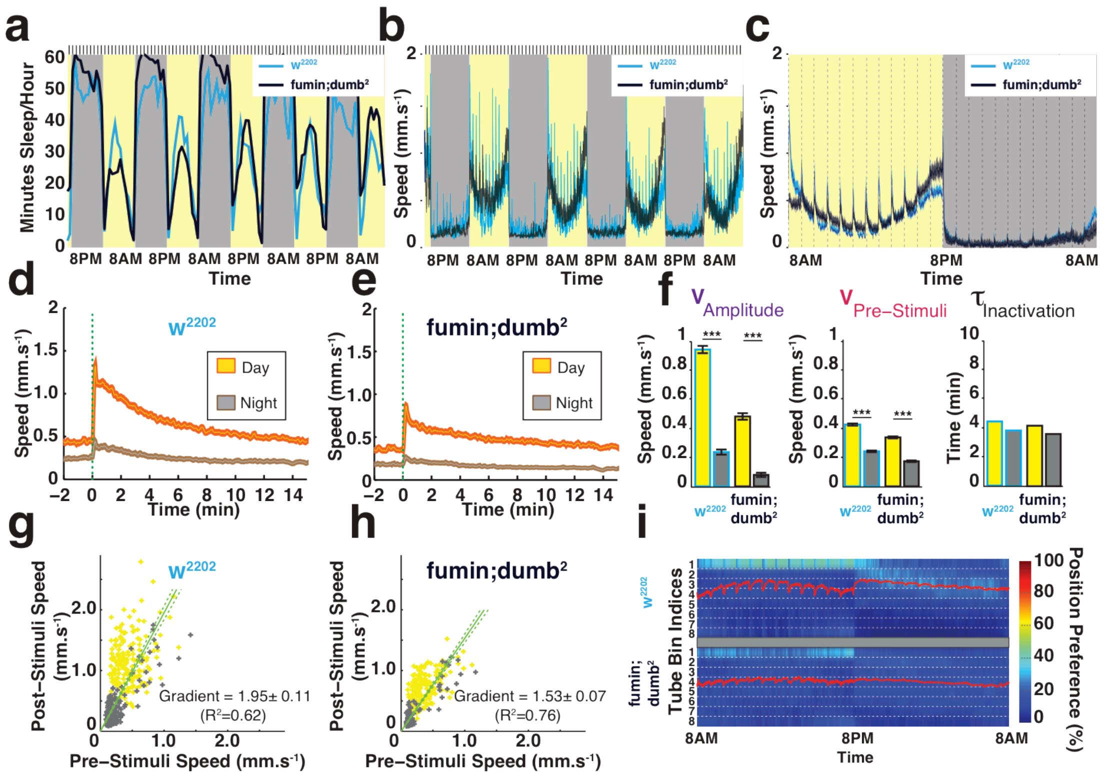

Fig. S10: Hourly arousal probing for w^{2202} and *fumin;dumb^2*. **a.** Average sleep profiles over several days (yellow) and nights (grey) for w^{2202} (blue) and *fumin;dumb^2* (black). Minutes of sleep per hour were determined by absolute location data, based on a 3mm movement threshold as in Fig. 2b. All flies were stimulated hourly (by vibrations, indicated by the black lines above the graph). N=68 for w^{2202} and N=66 for *fumin;dumb^2*. **b.** Average speed of w^{2202} (blue) and *fumin;dumb^2* (black) for the same set of flies, over the same time period as in A. The responses to the vibrations (black lines above the graph) are evident as spikes in the speed. **c.** Average speed data for day and night for the same set of flies. The vertical dashed lines indicate hourly vibrations testing for behavioral responsiveness. **d.** Average response (± SEM) for w^{2202}, for day (yellow) and night (grey). Green dashed line indicates timing of vibration stimulus (T=0). **e.** Average response (± SD) for *fumin;dumb^2*, for day (yellow) and night (grey). Green dashed line indicates timing of vibration stimulus (T=0). **f.** Response metrics for *fumin;dumb^2* (blue border) and *fumin;dumb^2* (black border) during the day (yellow) and night (grey). ***, *P*<0.001, by pair-wise 2-tailed t-test. **g.** Scatterplot of Pre-Stimuli speed *versus* Post-Stimuli speed, for individual day (yellow dots) and night (grey dots) events in w^{2202}. A regression of the data is shown, with associated slope and correlation indicated. **h.** Scatterplot of Pre-Stimuli speed *versus* Post-Stimuli speed, for individual day (yellow dots) and night (grey dots) events in *fumin;dumb^2*. A regression of the data is shown, with associated slope and correlation indicated. **i.** Heatmap of position preferences for w^{2202} and *fumin*;*dumb^2*, with average positions indicated by the red traces.

Table S1-4: Sleep Probing statistical support. List of statistical significance for each histogram in Figure 4b&c, using two-proportion z-test for the Reaction proportion (Tables S1 and S2) and pair-wise 2-tailed t-test for the Response Amplitude (Tables S3 and S4).

S1. w^{1118} Reaction Proportion Inter-Time Group Statistical Significance Levels

Reaction Proportion		W1118															
		1-5 (D)	6-10 (D)	11-15 (D)	16-20 (D)	21-25 (D)	26-30 (D)	31-35 (D)	36-40 (D)	1-5 (N)	6-10 (N)	11-15 (N)	16-20 (N)	21-25 (N)	26-30 (N)	31-35 (N)	36-40 (N)
W1118	1-5 (D)	N/S	N/S	N/S	0.05	N/S	0.01	N/S	N/S	10^(-5)	10^(-5)	10^(-10)	10^(-5)	10^(-10)	10^(-10)	10^(-10)	10^(-10)
	6-10 (D)	N/S	N/S	N/S	N/S	N/S	N/S	N/S	N/S	N/S	N/S	0.001	0.001	0.001	0.001	0.001	10^(-5)
	11-15 (D)	N/S	N/S	N/S	N/S	N/S	N/S	N/S	N/S	N/S	N/S	0.01	0.05	0.01	0.01	0.001	0.001
	16-20 (D)	0.05	N/S	N/S	N/S	N/S	N/S	N/S	N/S	N/S	N/S	N/S	N/S	N/S	0.05	0.05	0.01
	21-25 (D)	N/S	N/S	N/S	N/S	N/S	N/S	N/S	N/S	N/S	N/S	N/S	N/S	N/S	N/S	0.05	0.01
	26-30 (D)	0.01	N/S	N/S	N/S	N/S	N/S	0.05	N/S	N/S	N/S	N/S	N/S	N/S	N/S	N/S	0.01
	31-35 (D)	N/S	N/S	N/S	N/S	N/S	0.05	N/S	N/S	0.05	0.01	0.001	0.001	0.001	10^(-5)	10^(-5)	10^(-5)
	36-40 (D)	N/S	N/S	N/S	N/S	N/S	N/S	N/S	N/S	N/S	N/S	N/S	N/S	N/S	N/S	0.05	0.01
	1-5 (N)	10^(-5)	N/S	N/S	N/S	N/S	N/S	0.05	N/S	N/S	N/S	0.001	0.001	0.001	10^(-5)	10^(-5)	10^(-5)
	6-10 (N)	10^(-5)	N/S	N/S	N/S	N/S	N/S	0.01	N/S	N/S	N/S	0.05	0.05	0.05	0.01	0.01	0.001
	11-15 (N)	10^(-10)	0.001	0.01	N/S	N/S	N/S	0.001	N/S	0.001	0.05	N/S	N/S	N/S	N/S	N/S	0.05
	16-20 (N)	10^(-5)	0.001	0.05	N/S	N/S	N/S	0.001	N/S	0.001	0.05	N/S	N/S	N/S	N/S	N/S	0.05
	21-25 (N)	10^(-10)	0.001	0.01	N/S	N/S	N/S	0.001	N/S	0.001	0.05	N/S	N/S	N/S	N/S	N/S	N/S
	26-30 (N)	10^(-10)	0.001	0.01	0.05	N/S	N/S	10^(-5)	N/S	10^(-5)	0.01	N/S	N/S	N/S	N/S	N/S	N/S
	31-35 (N)	10^(-10)	0.001	0.001	0.05	0.05	N/S	10^(-5)	0.05	10^(-5)	0.01	N/S	N/S	N/S	N/S	N/S	N/S
	36-40 (N)	10^(-10)	10^(-5)	0.001	0.01	0.01	0.01	10^(-5)	0.01	10^(-5)	0.001	0.05	0.05	N/S	N/S	N/S	N/S
W2202	1-5 (D)	N/S	0.05	0.05	0.01	0.05	0.01	N/S	N/S	10^(-5)	10^(-5)	10^(-10)	10^(-10)	10^(-10)	10^(-10)	10^(-10)	10^(-10)
	6-10 (D)	0.05	N/S	N/S	N/S	N/S	N/S	N/S	N/S	N/S	0.05	10^(-5)	0.001	10^(-5)	10^(-5)	10^(-5)	10^(-5)
	11-15 (D)	0.001	N/S	N/S	N/S	N/S	N/S	0.05	N/S	N/S	N/S	0.001	0.01	0.01	0.001	0.001	10^(-5)
	16-20 (D)	0.001	N/S	N/S	N/S	N/S	N/S	0.05	N/S	N/S	N/S	0.01	0.05	0.05	0.01	0.01	0.001
	21-25 (D)	0.01	N/S	N/S	N/S	N/S	N/S	0.05	N/S	N/S	N/S	0.05	0.05	0.05	0.01	0.01	0.001
	26-30 (D)	N/S	N/S	N/S	N/S	N/S	N/S	N/S	N/S	N/S	N/S	0.01	0.05	0.01	0.01	0.01	0.001
	31-35 (D)	0.05	N/S	N/S	N/S	N/S	N/S	N/S	N/S	N/S	N/S	0.01	0.01	0.01	0.01	0.001	10^(-5)
	36-40 (D)	N/S	N/S	N/S	N/S	N/S	N/S	N/S	N/S	N/S	N/S	0.001	0.01	0.001	0.001	0.001	10^(-5)
	1-5 (N)	10^(-10)	0.001	0.05	N/S	N/S	N/S	0.001	N/S	0.001	N/S	N/S	N/S	N/S	N/S	0.05	0.001
	6-10 (N)	10^(-20)	10^(-10)	10^(-5)	0.001	0.01	0.01	10^(-5)	0.01	10^(-10)	10^(-5)	0.01	0.05	0.05	N/S	N/S	N/S
	11-15 (N)	10^(-20)	10^(-10)	10^(-5)	0.001	0.001	0.001	10^(-10)	0.01	10^(-20)	10^(-5)	0.001	0.01	0.01	0.05	N/S	N/S
	16-20 (N)	10^(-20)	10^(-10)	10^(-5)	0.001	0.001	0.001	10^(-10)	0.01	10^(-20)	10^(-5)	0.001	0.001	0.01	0.01	N/S	N/S
	21-25 (N)	10^(-50)	10^(-10)	10^(-10)	10^(-5)	0.001	0.001	10^(-10)	0.001	10^(-20)	10^(-10)	10^(-5)	0.001	0.001	0.001	0.05	N/S
	26-30 (N)	10^(-50)	10^(-10)	10^(-10)	10^(-5)	0.001	10^(-5)	10^(-10)	0.001	10^(-20)	10^(-10)	10^(-5)	10^(-5)	10^(-5)	0.001	0.01	N/S
	31-35 (N)	10^(-50)	10^(-10)	10^(-10)	10^(-5)	0.001	0.001	10^(-10)	0.001	10^(-20)	10^(-10)	10^(-5)	0.001	0.001	0.001	0.05	N/S
	36-40 (N)	10^(-50)	10^(-20)	10^(-10)	10^(-5)	10^(-5)	10^(-5)	10^(-10)	0.001	10^(-20)	10^(-10)	10^(-5)	10^(-5)	10^(-5)	10^(-5)	0.01	0.05

S2. w^{2202} Reaction Portion Inter-Time Group Statistical Significance Levels

Reaction Proportion		W2202															
		1-5 (D)	6-10 (D)	11-15 (D)	16-20 (D)	21-25 (D)	26-30 (D)	31-35 (D)	36-40 (D)	1-5 (N)	6-10 (N)	11-15 (N)	16-20 (N)	21-25 (N)	26-30 (N)	31-35 (N)	36-40 (N)
W1118	1-5 (D)	N/S	0.05	0.001	0.001	0.01	N/S	0.05	N/S	10^(-10)	10^(-20)	10^(-20)	10^(-20)	10^(-50)	10^(-50)	10^(-50)	10^(-50)
	6-10 (D)	0.05	N/S	N/S	N/S	N/S	N/S	N/S	N/S	0.001	10^(-10)	10^(-10)	10^(-10)	10^(-10)	10^(-10)	10^(-10)	10^(-20)
	11-15 (D)	0.05	N/S	N/S	N/S	N/S	N/S	N/S	N/S	0.05	10^(-5)	10^(-5)	10^(-5)	10^(-10)	10^(-10)	10^(-10)	10^(-10)
	16-20 (D)	0.01	N/S	N/S	N/S	N/S	N/S	N/S	N/S	N/S	0.001	0.001	0.001	10^(-5)	10^(-5)	10^(-5)	10^(-5)
	21-25 (D)	0.05	N/S	N/S	N/S	N/S	N/S	N/S	N/S	N/S	0.01	0.001	0.001	0.001	0.001	0.001	10^(-5)
	26-30 (D)	0.01	N/S	N/S	N/S	N/S	N/S	N/S	N/S	N/S	0.01	0.001	0.001	0.001	10^(-5)	0.001	10^(-5)
	31-35 (D)	N/S	N/S	0.05	0.05	0.05	N/S	N/S	N/S	0.001	10^(-5)	10^(-10)	10^(-10)	10^(-10)	10^(-10)	10^(-10)	10^(-10)
	36-40 (D)	N/S	N/S	N/S	N/S	N/S	N/S	N/S	N/S	N/S	0.01	0.01	0.01	0.001	0.001	0.001	0.001
	1-5 (N)	10^(-5)	N/S	N/S	N/S	N/S	N/S	N/S	N/S	0.001	10^(-10)	10^(-20)	10^(-20)	10^(-20)	10^(-20)	10^(-20)	10^(-20)
	6-10 (N)	10^(-5)	0.05	N/S	N/S	N/S	N/S	N/S	N/S	N/S	10^(-5)	10^(-5)	10^(-5)	10^(-10)	10^(-10)	10^(-10)	10^(-10)
	11-15 (N)	10^(-10)	10^(-5)	0.001	0.01	0.05	0.01	0.01	0.001	N/S	0.01	0.001	0.001	10^(-5)	10^(-5)	10^(-5)	10^(-5)
	16-20 (N)	10^(-10)	0.001	0.01	0.05	0.05	0.05	0.01	0.01	N/S	0.05	0.01	0.001	0.001	10^(-5)	0.001	10^(-5)
	21-25 (N)	10^(-10)	10^(-5)	0.01	0.05	0.05	0.01	0.01	0.001	N/S	0.05	0.01	0.01	0.001	10^(-5)	0.001	10^(-5)
	26-30 (N)	10^(-10)	10^(-5)	0.001	0.01	0.01	0.01	0.01	0.001	N/S	N/S	0.05	0.01	0.001	0.001	0.001	10^(-5)
	31-35 (N)	10^(-10)	10^(-5)	0.001	0.01	0.01	0.01	0.001	0.001	0.05	N/S	N/S	N/S	0.05	0.01	0.05	0.01
	36-40 (N)	10^(-10)	10^(-5)	10^(-5)	0.001	0.001	0.001	10^(-5)	10^(-5)	0.001	N/S	N/S	N/S	N/S	N/S	N/S	0.05
W2202	1-5 (D)	N/S	0.001	10^(-5)	0.001	0.001	0.05	0.05	0.05	10^(-20)	10^(-20)	10^(-20)	10^(-20)	10^(-50)	10^(-50)	10^(-50)	10^(-50)
	6-10 (D)	0.001	N/S	N/S	N/S	N/S	N/S	N/S	N/S	10^(-5)	10^(-10)	10^(-20)	10^(-20)	10^(-20)	10^(-20)	10^(-20)	10^(-20)
	11-15 (D)	10^(-5)	N/S	N/S	N/S	N/S	N/S	N/S	N/S	0.01	10^(-10)	10^(-10)	10^(-10)	10^(-10)	10^(-20)	10^(-10)	10^(-20)
	16-20 (D)	0.001	N/S	N/S	N/S	N/S	N/S	N/S	N/S	N/S	10^(-5)	10^(-5)	10^(-5)	10^(-10)	10^(-10)	10^(-10)	10^(-10)
	21-25 (D)	0.001	N/S	N/S	N/S	N/S	N/S	N/S	N/S	N/S	10^(-5)	10^(-5)	10^(-5)	10^(-10)	10^(-10)	10^(-10)	10^(-10)
	26-30 (D)	0.05	N/S	N/S	N/S	N/S	N/S	N/S	N/S	0.05	10^(-5)	10^(-5)	10^(-5)	10^(-5)	10^(-10)	10^(-5)	10^(-10)
	31-35 (D)	0.05	N/S	N/S	N/S	N/S	N/S	N/S	N/S	0.05	10^(-5)	10^(-5)	10^(-5)	10^(-10)	10^(-10)	10^(-10)	10^(-10)
	36-40 (D)	0.05	N/S	N/S	N/S	N/S	N/S	N/S	N/S	0.01	10^(-5)	10^(-10)	10^(-10)	10^(-10)	10^(-10)	10^(-10)	10^(-10)
	1-5 (N)	10^(-20)	10^(-5)	0.01	N/S	N/S	0.05	0.05	0.01	N/S	10^(-5)	10^(-5)	10^(-5)	10^(-10)	10^(-10)	10^(-10)	10^(-20)
	6-10 (N)	10^(-20)	10^(-10)	10^(-10)	10^(-5)	10^(-5)	10^(-5)	10^(-5)	10^(-5)	10^(-5)	N/S	N/S	N/S	0.05	0.01	0.05	0.001
	11-15 (N)	10^(-20)	10^(-20)	10^(-10)	10^(-5)	10^(-5)	10^(-5)	10^(-5)	10^(-10)	10^(-5)	N/S	N/S	N/S	N/S	0.05	N/S	0.01
	16-20 (N)	10^(-20)	10^(-20)	10^(-10)	10^(-5)	10^(-5)	10^(-5)	10^(-5)	10^(-10)	10^(-5)	N/S	N/S	N/S	N/S	N/S	N/S	N/S
	21-25 (N)	10^(-50)	10^(-20)	10^(-10)	10^(-10)	10^(-10)	10^(-5)	10^(-10)	10^(-10)	10^(-10)	0.05	N/S	N/S	N/S	N/S	N/S	N/S
	26-30 (N)	10^(-50)	10^(-20)	10^(-20)	10^(-10)	10^(-10)	10^(-10)	10^(-10)	10^(-10)	10^(-10)	0.01	0.05	N/S	N/S	N/S	N/S	N/S
	31-35 (N)	10^(-50)	10^(-20)	10^(-10)	10^(-10)	10^(-10)	10^(-5)	10^(-10)	10^(-10)	10^(-10)	0.05	N/S	N/S	N/S	N/S	N/S	N/S
	36-40 (N)	10^(-50)	10^(-20)	10^(-20)	10^(-10)	10^(-10)	10^(-10)	10^(-10)	10^(-10)	10^(-20)	0.001	0.01	N/S	N/S	N/S	N/S	N/S

S3. w^{1118} Amplitude Response Inter-Time Group Statistical Significance Levels

Response Amplitude		W2202															
		1-5 (D)	6-10 (D)	11-15 (D)	16-20 (D)	21-25 (D)	26-30 (D)	31-35 (D)	36-40 (D)	1-5 (N)	6-10 (N)	11-15 (N)	16-20 (N)	21-25 (N)	26-30 (N)	31-35 (N)	36-40 (N)
W1118	1-5 (D)	N/S	N/S	0.01	0.01	0.05	N/S	N/S	N/S	10^(-10)	10^(-10)	10^(-20)	10^(-20)	10^(-20)	10^(-20)	10^(-20)	10^(-20)
	6-10 (D)	N/S	N/S	0.05	0.05	N/S	N/S	N/S	N/S	0.001	10^(-5)	10^(-5)	10^(-5)	10^(-5)	10^(-5)	10^(-5)	10^(-5)
	11-15 (D)	N/S	N/S	N/S	N/S	N/S	N/S	N/S	N/S	0.05	0.01	0.001	0.001	0.001	0.001	0.001	0.001
	16-20 (D)	N/S	N/S	N/S	N/S	N/S	N/S	N/S	N/S	N/S	0.05	0.01	0.01	0.001	0.01	0.01	0.01
	21-25 (D)	N/S	N/S	N/S	N/S	N/S	N/S	N/S	N/S	N/S	0.05	0.05	0.05	0.01	0.01	0.01	0.01
	26-30 (D)	N/S	N/S	N/S	N/S	N/S	N/S	N/S	N/S	N/S	0.05	0.01	0.01	0.001	0.01	0.01	0.001
	31-35 (D)	N/S	N/S	0.05	0.05	N/S	N/S	N/S	N/S	0.01	0.001	0.001	0.001	0.001	0.001	0.001	0.001
	36-40 (D)	N/S	N/S	N/S	N/S	N/S	N/S	N/S	N/S	N/S	N/S	0.05	0.05	0.05	0.05	0.05	0.05
	1-5 (N)	N/S	N/S	N/S	N/S	N/S	N/S	N/S	N/S	10^(-5)	10^(-10)	10^(-10)	10^(-10)	10^(-20)	10^(-10)	10^(-10)	10^(-20)
	6-10 (N)	N/S	N/S	N/S	N/S	N/S	N/S	N/S	N/S	0.001	10^(-5)	10^(-10)	10^(-10)	10^(-10)	10^(-10)	10^(-10)	10^(-10)
	11-15 (N)	0.001	0.01	N/S	N/S	N/S	N/S	0.05	N/S	N/S	0.01	0.001	10^(-5)	10^(-5)	10^(-5)	10^(-5)	10^(-5)
	16-20 (N)	0.05	N/S	N/S	N/S	N/S	N/S	N/S	N/S	N/S	0.01	0.001	0.001	10^(-5)	0.001	0.001	0.001
	21-25 (N)	0.05	N/S	N/S	N/S	N/S	N/S	N/S	N/S	N/S	0.01	0.001	0.001	10^(-5)	0.001	0.001	0.001
	26-30 (N)	0.01	0.05	N/S	N/S	N/S	N/S	0.05	0.05	N/S	N/S	0.05	0.01	0.001	0.01	0.01	0.01
	31-35 (N)	0.001	0.001	0.05	0.05	0.05	0.05	0.01	0.01	N/S	N/S	N/S	N/S	0.05	N/S	N/S	N/S
	36-40 (N)	10^(-5)	10^(-5)	0.01	0.01	0.01	0.01	0.001	0.001	N/S	N/S	N/S	N/S	0.05	N/S	N/S	0.05
W2202	1-5 (D)	N/S	N/S	0.01	0.01	N/S	N/S	N/S	N/S	10^(-10)	10^(-10)	10^(-20)	10^(-20)	10^(-50)	10^(-20)	10^(-20)	10^(-20)
	6-10 (D)	N/S	N/S	N/S	0.05	N/S	N/S	N/S	N/S	10^(-5)	10^(-10)	10^(-10)	10^(-10)	10^(-20)	10^(-10)	10^(-10)	10^(-20)
	11-15 (D)	0.01	N/S	N/S	N/S	N/S	N/S	N/S	N/S	0.01	0.001	10^(-5)	10^(-5)	10^(-10)	10^(-5)	10^(-5)	10^(-5)
	16-20 (D)	0.01	0.05	N/S	N/S	N/S	N/S	N/S	N/S	0.05	0.001	10^(-5)	10^(-5)	10^(-10)	10^(-5)	10^(-5)	10^(-5)
	21-25 (D)	N/S	N/S	N/S	N/S	N/S	N/S	N/S	N/S	0.05	0.001	10^(-5)	10^(-5)	10^(-5)	10^(-5)	10^(-5)	10^(-5)
	26-30 (D)	N/S	N/S	N/S	N/S	N/S	N/S	N/S	N/S	0.05	0.001	0.001	0.001	10^(-5)	10^(-5)	10^(-5)	10^(-5)
	31-35 (D)	N/S	N/S	N/S	N/S	N/S	N/S	N/S	N/S	0.001	0.001	10^(-5)	10^(-5)	10^(-5)	10^(-5)	10^(-5)	10^(-5)
	36-40 (D)	N/S	N/S	N/S	N/S	N/S	N/S	N/S	N/S	0.001	10^(-5)	10^(-5)	10^(-5)	10^(-10)	10^(-5)	10^(-5)	10^(-5)
	1-5 (N)	10^(-10)	10^(-5)	0.01	0.05	0.05	0.05	0.001	0.001	N/S	N/S	0.001	0.001	10^(-5)	0.001	10^(-5)	10^(-5)
	6-10 (N)	10^(-10)	10^(-10)	0.001	0.001	0.001	0.001	0.001	10^(-5)	N/S	N/S	N/S	N/S	0.01	0.05	0.05	0.05
	11-15 (N)	10^(-20)	10^(-10)	10^(-5)	10^(-5)	10^(-5)	0.001	10^(-5)	10^(-5)	0.001	N/S	N/S	N/S	N/S	N/S	N/S	N/S
	16-20 (N)	10^(-20)	10^(-10)	10^(-5)	10^(-5)	10^(-5)	0.001	10^(-5)	10^(-5)	0.001	N/S	N/S	N/S	N/S	N/S	N/S	N/S
	21-25 (N)	10^(-50)	10^(-20)	10^(-10)	10^(-10)	10^(-5)	10^(-5)	10^(-5)	10^(-10)	10^(-5)	0.01	N/S	N/S	N/S	N/S	N/S	N/S
	26-30 (N)	10^(-20)	10^(-10)	10^(-5)	10^(-5)	10^(-5)	10^(-5)	10^(-5)	10^(-5)	0.001	0.05	N/S	N/S	N/S	N/S	N/S	N/S
	31-35 (N)	10^(-20)	10^(-10)	10^(-5)	10^(-5)	10^(-5)	10^(-5)	10^(-5)	10^(-5)	10^(-5)	0.05	N/S	N/S	N/S	N/S	N/S	N/S
	36-40 (N)	10^(-20)	10^(-20)	10^(-5)	10^(-5)	10^(-5)	10^(-5)	10^(-5)	10^(-5)	10^(-5)	0.05	N/S	N/S	N/S	N/S	N/S	N/S

S4. w^{2202} Amplitude Response Inter-Time Group Statistical Significance Levels

Response Amplitude		W1118															
		1-5 (D)	6-10 (D)	11-15 (D)	16-20 (D)	21-25 (D)	26-30 (D)	31-35 (D)	36-40 (D)	1-5 (N)	6-10 (N)	11-15 (N)	16-20 (N)	21-25 (N)	26-30 (N)	31-35 (N)	36-40 (N)
W1118	1-5 (D)	N/S	N/S	N/S	N/S	N/S	0.05	N/S	N/S	N/S	N/S	0.001	0.01	0.01	0.001	10^(-5)	10^(-5)
	6-10 (D)	N/S	N/S	N/S	N/S	N/S	N/S	N/S	N/S	N/S	N/S	0.01	0.05	0.05	0.01	0.001	0.001
	11-15 (D)	N/S	N/S	N/S	N/S	N/S	N/S	N/S	N/S	N/S	N/S	N/S	N/S	N/S	N/S	0.05	0.01
	16-20 (D)	N/S	N/S	N/S	N/S	N/S	N/S	N/S	N/S	N/S	N/S	N/S	N/S	N/S	N/S	N/S	N/S
	21-25 (D)	N/S	N/S	N/S	N/S	N/S	N/S	N/S	N/S	N/S	N/S	N/S	N/S	N/S	N/S	N/S	N/S
	26-30 (D)	0.05	N/S	N/S	N/S	N/S	N/S	N/S	N/S	N/S	N/S	N/S	N/S	N/S	N/S	N/S	N/S
	31-35 (D)	N/S	N/S	N/S	N/S	N/S	N/S	N/S	N/S	N/S	N/S	0.05	0.05	0.05	0.05	0.01	0.01
	36-40 (D)	N/S	N/S	N/S	N/S	N/S	N/S	N/S	N/S	N/S	N/S	N/S	N/S	N/S	N/S	N/S	N/S
	1-5 (N)	N/S	N/S	N/S	N/S	N/S	N/S	N/S	N/S	N/S	N/S	0.05	N/S	N/S	0.05	0.001	10^(-5)
	6-10 (N)	N/S	N/S	N/S	N/S	N/S	N/S	N/S	N/S	N/S	N/S	0.05	N/S	N/S	0.05	0.01	0.001
	11-15 (N)	0.001	0.01	N/S	N/S	N/S	N/S	0.05	N/S	0.05	0.05	N/S	N/S	N/S	N/S	N/S	0.05
	16-20 (N)	0.01	0.05	N/S	N/S	N/S	N/S	0.05	N/S	N/S	N/S	N/S	N/S	N/S	N/S	N/S	0.05
	21-25 (N)	0.01	0.05	N/S	N/S	N/S	N/S	0.05	N/S	N/S	N/S	N/S	N/S	N/S	N/S	N/S	0.05
	26-30 (N)	0.001	0.01	N/S	N/S	N/S	N/S	0.05	N/S	0.05	0.05	N/S	N/S	N/S	N/S	N/S	N/S
	31-35 (N)	10^(-5)	0.001	0.05	N/S	N/S	N/S	0.01	N/S	0.001	0.01	N/S	N/S	N/S	N/S	N/S	N/S
	36-40 (N)	10^(-5)	0.001	0.01	N/S	N/S	N/S	0.01	N/S	10^(-5)	0.001	0.05	0.05	0.05	N/S	N/S	N/S
W2202	1-5 (D)	N/S	N/S	N/S	N/S	N/S	N/S	N/S	N/S	N/S	N/S	0.001	0.05	0.05	0.01	0.001	10^(-5)
	6-10 (D)	N/S	N/S	N/S	N/S	N/S	N/S	N/S	N/S	N/S	N/S	0.01	N/S	N/S	0.05	0.001	10^(-5)
	11-15 (D)	0.01	0.05	N/S	N/S	N/S	N/S	0.05	N/S	N/S	N/S	N/S	N/S	N/S	N/S	0.05	0.01
	16-20 (D)	0.01	0.05	N/S	N/S	N/S	N/S	0.05	N/S	N/S	N/S	N/S	N/S	N/S	N/S	0.05	0.01
	21-25 (D)	0.05	N/S	N/S	N/S	N/S	N/S	N/S	N/S	N/S	N/S	N/S	N/S	N/S	N/S	0.05	0.01
	26-30 (D)	N/S	N/S	N/S	N/S	N/S	N/S	N/S	N/S	N/S	N/S	N/S	N/S	N/S	N/S	0.05	0.01
	31-35 (D)	N/S	N/S	N/S	N/S	N/S	N/S	N/S	N/S	N/S	N/S	0.05	N/S	N/S	0.05	0.01	0.001
	36-40 (D)	N/S	N/S	N/S	N/S	N/S	N/S	N/S	N/S	N/S	N/S	N/S	N/S	N/S	0.05	0.01	0.001
	1-5 (N)	10^(-10)	0.001	0.05	N/S	N/S	N/S	0.01	N/S	10^(-5)	0.001	N/S	N/S	N/S	N/S	N/S	N/S
	6-10 (N)	10^(-10)	10^(-5)	0.01	0.05	0.05	0.05	0.001	N/S	10^(-10)	10^(-5)	0.01	0.01	0.01	N/S	N/S	N/S
	11-15 (N)	10^(-20)	10^(-5)	0.001	0.01	0.05	0.01	0.001	0.05	10^(-10)	10^(-10)	0.001	0.001	0.001	0.05	N/S	N/S
	16-20 (N)	10^(-20)	10^(-5)	0.001	0.01	0.05	0.01	0.001	0.05	10^(-10)	10^(-10)	10^(-5)	0.001	0.001	0.01	N/S	N/S
	21-25 (N)	10^(-20)	10^(-5)	0.001	0.001	0.01	0.001	0.001	0.05	10^(-20)	10^(-10)	10^(-5)	10^(-5)	10^(-5)	0.001	0.05	0.05
	26-30 (N)	10^(-20)	10^(-5)	0.001	0.01	0.01	0.01	0.001	0.05	10^(-10)	10^(-10)	10^(-5)	0.001	0.001	0.01	N/S	N/S
	31-35 (N)	10^(-20)	10^(-5)	0.001	0.01	0.01	0.01	0.001	0.05	10^(-10)	10^(-10)	10^(-5)	0.001	0.001	0.01	N/S	N/S
	36-40 (N)	10^(-20)	10^(-5)	0.001	0.01	0.01	0.001	0.001	0.05	10^(-20)	10^(-10)	10^(-5)	0.001	0.001	0.01	N/S	0.05
